# Environmental Impacts of Cultured Meat: A Cradle-to-Gate Life Cycle Assessment

**DOI:** 10.1021/acsfoodscitech.4c00281

**Published:** 2024-12-30

**Authors:** Derrick Risner, Patrick Negulescu, Yoonbin Kim, Cuong Nguyen, Justin B. Siegel, Edward S. Spang

**Affiliations:** 1 Department of Food Science and Technology, University of California, Davis, California 95616, United States; 2 Department of Chemical Engineering, University of California, Davis, California 95616, United States; 3 Division of Agriculture and Natural Resources, University of California, Holtville, California 92250, United States; 4 Genome Center, University of California, Davis, California 95616, United States; 5 Departments of Chemistry, Biochemistry and Molecular Medicine, University of California, Davis, California 95616, United States; 6 Innovation Institute for Food and Health, University of California, Davis, California 95616, United States; 7 USDA, AI Institute for Next Generation Food Systems (AIFS), University of California, Davis, California 95616, United States

**Keywords:** animal cell-based meat, cultured meat, environment, life cycle assessment

## Abstract

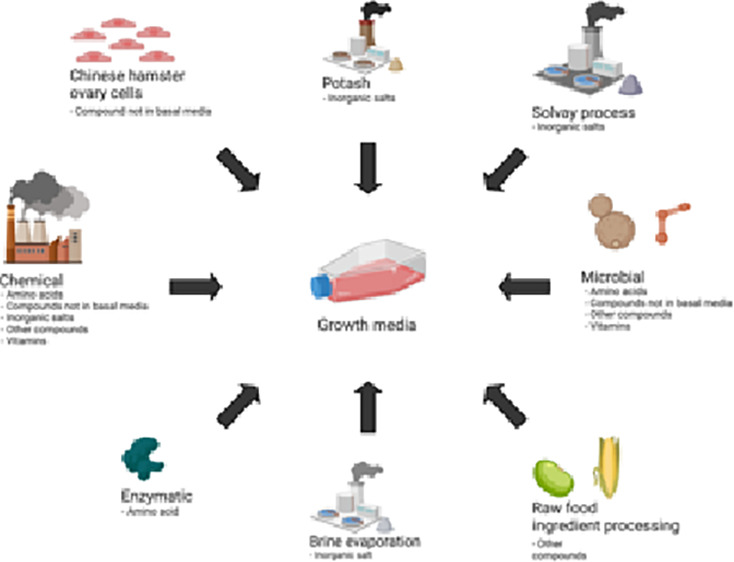

Interest in animal cell-based meat (ACBM) as an environmentally conscious replacement for livestock production has been increasing; however, a life cycle assessment (LCA) for the existing production methods of ACBM has not been conducted. Currently, ACBM products are being produced at a small scale, but ACBM companies are intending to scale-up production. Updated findings from recent technoeconomic assessments (TEAs) of ACBM were utilized to perform an LCA of near-term ACBM production. A scenario analysis was conducted utilizing the metabolic requirements examined in the TEAs of ACBM, and a purification factor was utilized to account for growth medium component processing. The results indicate that the environmental impact of near-term ACBM production has the potential to be significantly higher than beef if a highly refined growth medium is utilized for ACBM production. This study highlights the need to develop a sustainable animal cell growth medium that is optimized for high-density animal cell proliferation for ACBM to generate positive economic and environmental benefits.

## Introduction

Producing sustainable and healthy protein is emerging as one of the key challenges of our century, especially considering estimates that the global demand for protein will double by 2050.^1^ The urgency of this issue is accentuated by findings from a recent Rockefeller Foundation study examining the “true cost of food,” which revealed roughly a one-to-one cost ratio between what consumers spend on food in America and the consequent environmental damage.^2^ This alarming economic-environmental parity demands a radical transformation of our food production systems. Without the rapid and widespread adoption of sustainable protein solutions, we risk inflating the hidden environmental costs to dire levels, jeopardizing not only economic stability but also the health of our planet.

Historically, the highest quality and most desired sources of protein are derived from animal sources.^1^ Global meat production has increased from 71 million metric tons in 1961 to 337 million metric tons in 2020, though the consumption of different meat sources is highly regionalized.^3,4^ In 2020, beef and buffalo meat accounted for ∼22% of global meat production, and poultry and pork accounted for ∼39 and ∼32% of worldwide meat production, respectively.^3,4^ While there are many new transformative technologies being deployed to further increase meat production while minimizing environmental impact,^5−7^ there is agreement that the production of additional high-quality protein that meets consumer cultural demands is needed. One of the most futuristic concepts for additional protein production is cultured ACBM. While there are many recent exciting technological advances in the development of ACBM production, there has yet to be a “cradle-to-gate” LCA that examines what is currently achievable for ACBM production and highlights the technical challenges related to making this potential product less environmentally impactful.

Briefly, the core concept of cultured meat production is that animal cells such as pluripotent stem cells can be proliferated in industrial-scale bioreactors (>1000 L), differentiated into a variety of cell types (e.g., adipocytes, myotubes, and fibroblasts), and then processed for human consumption in place of conventionally produced meat.^8,9^ Currently, Singapore, United States, and Israel have supplied regulatory approval for commercial ACBM products for human consumption.^10^ These ACBM products were produced for high-end dining, albeit at the time of this writing, no cultured meat products are produced at a large-enough scale to be considered broadly commercially available. The lack of product can be attributed to a number of challenges faced by ACBM companies with the economic feasibility and environmental impact being tightly linked to one another and additional challenges such as nutrition, public perception, and taste being on the horizon.

Despite the lack of a full LCA, the technology is receiving significant investments to explore technological feasibility, with estimates at the time of writing this article totaling more than $3 billion.^10^ Given the magnitude of the investment and the emergence of initial products from a novel technology in the marketplace, it is imperative to conduct a comprehensive LCA. This analysis is crucial to identify and address key challenges necessary for achieving a favorable environmental impact, particularly in comparison to traditional protein sources.

When the top three livestock production systems are examined from an environmental perspective, beef is the most impactful per kilogram, though this value varies significantly by production system.^11^ The environmental impact of beef production includes greenhouse gas emissions (GHGs) from enteric fermentation and manure, nutrient loading in the nitrogen and phosphorus cycles, reduction in biodiversity from overgrazing, and deforestation from land-use change.^12,13^

Multiple LCAs have examined different beef production systems with global warming potential, or GWP (kg of carbon dioxide equivalent, CO_2_e), the most commonly utilized environmental metric.^14^ The environmental impacts are quantified based on the functional unit of the beef product (e.g., live weight, carcass weight, or boneless meat), which varies across studies. For example, skeletal muscle is only one product produced from a slaughter facility.^15^ Approximately 78.3% mass of the animal is utilized as primal cuts of meat (37.8%), rendering products (32.8%), raw hide (4.9%), and offal (3.2%) in the United States and Canada.^15^ A 2015 review of beef LCAs reported a range of 7.6 (live weight) to 29.7 kg (carcass weight) of CO_2_e per kg of beef.^14^

The reported values in the literature vary significantly due to differences in functional unit, as mentioned above, but also by the production system (e.g., origin of calf, organic vs nonorganic, and type of diet) and geographic location.^14^ A study that examined the environmental impact of multiple foods at the retail level indicated GHG emissions ranged from 9.6 to 432 kg of CO_2_e for each kilogram of fat and bone-free meat and edible offal (FBFMO) produced.^11^ The reported GHG emissions from meat produced from a beef herd (cattle raised with primary purpose of meat production) ranged from 35 to 432 kg of CO_2_e per kg of FBFMO with mean and median values of 99.5 and 60.4 kg of CO_2_e per kg of FBFMO, respectively. The GHG emissions from FBFMO produced from dairy herds ranged from 9.6 to 73.9 kg of CO_2_e per kg of FBFMO, with mean and median values of 33.4 and 34.1 kg of CO_2_e per kg of FBFMO.^11^ This difference in the kg of CO_2_e per kg of FBFMO for beef and dairy herds is due to the allocation of environmental impact across both meat and dairy products in the dairy herds. The relative closeness of the mean and median indicate fewer outliers for dairy herd-produced FBFMO. Due to the potential environmental impacts of increased beef production and animal welfare concerns, beef production has been identified as a large-scale food production system that could be augmented, modified, or significantly curtailed.^16,17^

To model a robust life cycle analysis for agricultural systems, it is generally necessary to start from a current or proposed commercial-scale product system. In the case of ACBM, there are no commercial-scale mass production systems in operation as of writing this article. However, over the past few years, three independent groups have developed models based on adopting practices derived from the well-established fermentation industry and then linking these models to envisioned future systems bounded by biological and physical limitations.

The first TEA for ACBM was published by Risner et al., which examined the core capital and operating expenditures required to produce cultured meat at scale.^8^ Given the uncertainty of auxiliary processes (e.g., scaffolding and product forming or shaping), the TEA focused only on the core cell proliferation and differentiation processes in production-scale bioreactors. The bioreactors represented the system capital costs, while the variable operating expenditures included ingredients, raw materials, some utilities, and labor costs. The “Risner et al. TEA” included Essential 8 (E8) as the animal cell growth medium in the model. E8 is a defined growth medium designed for stem cell research and had been previously suggested as a growth medium that could be scaled and slightly modified for the industrial production of cultured meat.^18−20^ The authors assume that the use of E8 or a similar refined growth medium is necessary as *in vitro* animal cells are sensitive to media impurities, and the growth medium will likely need to be optimized for individual cell lines.

Given the uncertainty inherent to modeling an emerging technology, the Risner et al. TEA included an assessment of four potential scenarios to produce 122 million kg of wet cells of cultured meat (i.e., 36.6 million kg of dry cells or 25.62 million kg of protein). Scenarios 1 and 4 represented “bookend” scenarios where scenario 1 represented the initial state of cultured meat production mirroring the economics of early proof-of-concept demonstrations, and scenario 4 represented pushing the system to the physical and biological limits of the bioreactor (thus providing a theoretical boundary case but not an operationally realistic scenario for actual cultured meat production). Scenarios 2 and 3 represented “midpoint” scenarios where a few particularly critical cost hurdles were overcome.

Shortly after the Risner et al. TEA was published, a more complete TEA commissioned by Open Philanthropy and conducted by Davis Humbird was peer reviewed and published in *Biotechnology and Bioengineering*.^9^ The “Humbird TEA” examined a complete production system and included all of the equipment that would be necessary to produce 100 million kg of cultured meat per year, utilizing chemical engineering scaling equations to estimate costs at scale. It examined a more simplified growth medium with commodity level pricing and limited refinement of the carbon source.

In 2022, an additional TEA (“Negulescu TEA”) was conducted, which modeled bioreactor systems with volumes greater than what has been achieved for pharmaceutical animal cell propagation (>25,000 L).^21^ The modeled system included a 41,000 L stir tank bioreactor, a 211,000 L stir tank bioreactor system, and a 262,000 L airlift bioreactor. The Negulescu TEA also included chemical engineering scaling equations to estimate costs at scale.

These TEAs collectively underscored six major technical and economic challenges for the development of this nascent protein production system previously identified by industry professionals:^22^

1. Cells lines would need to be developed with properties superior to the best cell lines currently used in current biopharmaceutical practice (i.e., growing to higher cell concentrations by limiting waste product or osmotic inhibition at a level greater than previously developed animal cell lines).
2. Bioreactors would have to be operated at much larger scales than current pharmaceutical production for economic viability.
3. Bioreactors would need to be operated under conditions significantly outside of the range of current biopharmaceutical engineering rules of thumb to effectively scale the production process (e.g., using a higher a scaling factor than convention (4×) between seed train bioreactors).
4. The system would require aseptic operation (including viral exclusion) at a very large scale beyond the current practice to avoid contamination and potential batch loss.
5. Low-cost sources of amino acids of suitable quality for animal cell proliferation and differentiation would need to be developed.
6. The amino acid supply chain would need to be scaled up far beyond the current manufacturing volumes.

Developing solutions to these challenges is essential to both the economic success of cultured meat and reducing the environmental impact of these potential products. The especially critical challenge is to successfully optimize cell growth while simultaneously reducing the complexity and cost of the growth medium. High cell concentrations (>1 × 10^8^ cells/ml) have been achieved in lab-scale perfusion bioreactors (∼2–10 L) utilizing highly refined growth mediums (serum-free UltraCULTURE, supplemented CHO CD XP with hydrolysate and supplemented PF-CHO Liquid Soy medium).^23,24^ However, similar concentrations have not been achieved using a less-refined growth medium or production-scale stirred tank bioreactors.^25^ Researchers have also explored utilizing filtered (0.22 μm filter) food-grade growth medium components and found that animal cell proliferation was possible.^26^ However, it is important to note that the growth medium was supplemented with fetal bovine serum (at a level of 10–20%) and the cells were grown in 60 mm dishes, which would not be a scalable solution for the industrial production of cultured meat.^26^ Plant hydrolysates have also been used to supplement amino acids in animal cell growth mediums with some success,^24,27^ but not as the sole source of amino acids for animal cell culture. These issues highlight the economic challenges that ACBM companies are confronted with, and many of these challenges are mirrored when considering the environmental impact of the ACBM products. Additional challenges related to nutrition, public perception, and taste have also been identified; however, these are likely be a higher priority if the economic uncertainty diminishes.^28^

A number of existing studies have suggested that the potential environmental impact of producing cultured meat would be less than conventionally produced beef.^29−31^ However, these studies were not based on any of the more advanced TEA systems that have recently been modeled. The LCA process models in these studies are often based on cultured meat production systems that drastically depart from the core assumptions of realistic near-term ACBM production. Furthermore, a careful review and gap analysis of some these some of these studies suggested the need for further environmental assessment.^32^

For example, an often-cited LCA of cultured meat production estimates 1.9–2.2 kg CO_2_eq GHG emissions and 26–33 MJ energy consumption per kg of cultured meat produced.^29^ However, this assessment is based on utilizing cyanobacteria hydrolysate as an ingredient for the growth medium to feed the animal cells. However, this is not a feedstock that is currently used for cultured meat production nor is it one that is near feasibility given the current technical challenges of cultured meat production. An amendment to the original study was later published that acknowledged this limitation of the proposed production system.^30^ The published amendment went on to examine different scenarios with different feedstocks and bioreactor combinations, but the authors also acknowledged the high levels of uncertainty inherent to these untested approaches.^30^

Another cultured meat LCA that provided an increased level of detail was published in 2015.^31^ However, a close examination of the assumptions reveals some significant limitations in terms of modeling a production line without evidence of feasibility.^33^ The modeled process assumes the use of soy protein hydrolysate as an amino acid source, neglects to apply specific consumption rates to estimate the utilization of basal media and amino acids, and proposes the use of corn starch microcarriers for cell proliferation.^31^ These layered assumptions combine to create a model that is not an accurate representation of current or near-term cultured meat production.

Similarly, a recent *ex ante* LCA of cultured meat relied entirely on highly uncertain projections of future ACBM production in 2030, which included broad assumptions about significant technological advances in ACBM production processes as well as the upstream supply chains for amino acids, growth factors, vitamins, salts, and other components.^34^ It was assumed that a soy hydrolysate would be utilized for 75% of the required amino acids, and the growth medium components would be food-or-feed grade for animal cell culture.^34^ Furthermore, it was assumed that wastewater would largely be recycled (75%) and would only require a level of processing similar to wastewater treatment at a potato starch production facility.^34^ The authors are unaware of any studies that would validate these assumptions to generate the high levels of animal cell proliferation and density necessary for the economically viable production of cultured meat.

In the 2022 Tuomisto et al. LCA, the authors examined the use of perfusion bioreactors as a production method. Perfusion bioreactors constantly feed fresh growth medium into the bioreactor while simultaneously removing the spent-cell-free growth medium. Unfortunately, this operational strategy often leads to a lower titer when compared to fed-batch operations and has been modeled to be less economically feasible as well.^9,35,36^ Furthermore, the study utilizes the environmental impact of urea production as a proxy for the production of fetal bovine serum (FBS).^37^ This likely skews the results toward a reduced environmental impact as urea is much simpler to produce than FBS. The current processing/supply chain of FBS is a multistep process (abattoir, blood collection, serum separation, raw serum freezing, raw FBS selection, thawing, pooling and prefiltration, aseptic filling, packaging, labeling, finished production, final labeling, final production freezing, storage, optional gamma irradiation, distributor then end-user)^38^ and is likely to be highly resource intensive.

An LCA of a hybrid cultured meat/plant/fungi burger product was conducted recently, but the model is not transparent as the process model relies on confidential data from an industry partner, SCiFi foods.^39^ Thus, there is no visibility into the mass or quantity of the growth medium components utilized in hybrid LCA. This is critical information for product assessment and study reproducibility as growth media costs have been the economically limiting factor in previous TEAs of cultured meat.^8,9,21^

A 2019 study examined ACBM in the context of long-term climate modeling.^40^ This study utilized existing assessments of both beef and ACBM to determine the long-term (∼1000 year) global warming implication for the mass production of both these products. A key aspect of the study was it examined how different emission types (ex. CO_2_ vs CH_4_) played a role in the long-term global warming implications of these meat production systems. In some scenarios, cultured meat production increased global temperatures more than beef production and this was largely due to the limited atmospheric life of methane.^40^

In summary, existing estimates of the environmental impact of cultured meat production are marked by significant uncertainty due to their dependence on speculative models of future production systems without solid TEAs. To bridge this knowledge gap and accurately discern the environmental implications of cultured meat production, we have performed a detailed cradle-to-gate LCA grounded in peer-reviewed TEAs specific to cultured meat.^8,9,21^ This approach has enabled us to directly correlate economic and environmental impacts, facilitating a critical examination of the essential factors that future products must meet to be commercially viable and environmentally competitive with conventional systems.

## Materials and Methods

The cultured meat LCA was conducted following the ISO 14040 and 14044 standards to estimate the environmental impact of production, including definition of goal and scope, life cycle inventory analysis, life cycle impact assessment, and interpretation.^41,42^ A combination of peer-reviewed literature, OpenLCA v.1.10 software, existing databases, stoichiometric calculations, and engineering judgment was utilized to model the production system.

A system boundary was set at the cradle (raw material extraction) to the cultured meat production facility gate. Given that this LCA stops at the cultured meat production facility gate, it does not include product losses, cold storage, transportation, and other environmental impacts associated with the retail sale of beef. In accordance with the ISO 14040 and 14044 standards, we have chosen the functional unit of a single kilogram of cultured meat (wet basis) to allow for comparison with a similar conventionally produced ground beef product or other cultured meat products.

We first assessed the production of the growth media and then utilized the results of this analysis to inform our model of cultured meat production. In fact, the majority of our life cycle inventory (LCI) focuses on the growth media with the remaining inputs specific to cultured meat production, including energy and water use. Several previous TEAs have identified the growth medium cost as core economic challenge for industrial cultured meat production.^8,9,21^ Thus, understanding the life cycle impacts to produce growth media currently used in cultured meat production is essential in the analysis of the cradle-to-production gate environmental impact of cultured meat. We also considered two scenarios of growth media inputs: our core analysis of food- or feed-grade ingredients and a secondary estimation of pharmaceutical-grade ingredients. Detailed data are less available on pharmaceutical-grade components, so we used a multiplying factor^43^ to provide a rough estimate of how the food/feed and pharmaceutical pathways of cell media production might influence environmental impact.

Finally, for our process model, we generated three additional scenarios based on assumptions regarding the amount of growth medium required for cultured meat production. We define the details in much greater detail in a later section, but at a high level, we generate three scenarios: (1) glucose as a limiting factor “normal” cell metabolism, (2) amino acids as a limiting factor for protein synthesis, and (3) for an “enhanced” cell metabolism (i.e., less glucose required).^9,21^

### LCI

The LCI analysis predominantly included tracking all the inputs to generate the growth media, followed by tracking the additional inputs (e.g., water and energy) for cultured meat production.

#### Growth Media

E8 is a defined growth medium that has been utilized and promoted as a viable growth medium for stem cells and cultured meat production.^18,20,44,45^ The E8 growth medium was originally designed for researchers studying human-induced pluripotent stem cells and embryonic stem cells. E8 was formulated as a consistent, defined medium to improve experiment reproducibility, but was not originally designed as a growth medium for industrial cell biomass production.^20^ A derivative product of E8, Beefy-9 (B9), was also assessed for comparison. B9 is currently not widely used for animal culture; however, it has been designed with cultured meat in mind.^46^ Additionally, an antibiotic-free version of B9 was assessed to understand the impact of the addition of antibiotics.

E8 and B9 are largely composed of Dulbecco’s modified Eagle medium/Hams’ F12 (DMEM/F12) basal medium, which is widely used for animal cell culture along with seven other ingredients, including 2-phospho-l-ascorbic acid trisodium salt, insulin, transferrin, sodium selenite, fibroblast growth factor-2 (FGF-2), transforming growth factor beta (TGF-β), and additional sodium bicarbonate (E8 only) (48). B9 contains the same components as E8 with the additional components of neuregulin, ultrapure water, antibiotics/antimycotics, and recombinant albumin.^46^

Production process information for each media component was initially searched for in the ecoinvent (v.3.8) LCI database.^47^ If available in ecoinvent, then the material and energy input flows were tracked utilizing these data sets.^47^ If the initial production process information was not available in ecoinvent, then other literature sources and calculations were utilized to estimate material inputs and outputs (see the following section, supplemental tables/figures, Appendix A–H). A limit of 0.1 kg of reactant or precursor per kilogram of input was deemed the minimum limit to continue to track a component. For the sake of this study, precursor refers to a material/chemical used to produce an ingredient in the E8/B9 growth medium (e.g., starch hydrolysate is a precursor to glucose).

Ecoinvent’s global data sets were utilized throughout the LCI to limit the effect of geographic variation. The ecoinvent database can be examined with five different settings (undefined, allocation (cutoff by classification), allocation at the point of substitution, substitution (consequential, long-term), and allocation (cutoff, EN15804)), which unlink or link data sets using several different methodologies. The database search was configured to “undefined” to maximize the LCI analysis transparency.^47^ An undefined system model unlinks unit processes and allows for multiple outputs from each unit process.

The flows and processes were then imported and configured in the OpenLCA software, which tracks inputs/outputs for a product system. The estimated material and energy flows should be considered nonexhaustive as the industrial production processes for some media components (e.g., 4-(2-hydroxyethyl)-1-piperazineethanesulfonic acid (HEPES) and lipoic acid production) were excluded, and other E8/B9 component production processes were only partially represented as a result of gaps in the available data.

The methods, calculations, limitations, and assumptions for the LCA model are further elaborated in the subsequent sections, including additional detail on each of the individual components of the E8/B9 media as organized into eight categories of the production method.^18,20^ It should also be noted that the reported E8/B9 component production processes do not represent the production of cell culture-grade materials. Production of more highly purified cell culture-grade materials requires additional resources, and this is addressed in the Scenario Analysis section.

##### Raw Food Ingredients

Corn was assumed to be the source for glucose as it is widely utilized for biorefining and food/beverage production in the United States.^48^ Cottonseed oil production was used to estimate linoleic acid production given its alignment with the cottonseed oil fatty acid profile.^49^ Ecoinvent data sets were used to estimate the material flow for both glucose and linoleic acid. Appendix A provides details on the calculations and procedures utilized to determine the material flows of glucose and linoleic acid.

##### Microbial Fermentation Products

Components of E8/B9 which are, or have potential to be, produced via microbial fermentation were identified (Tables A1.0 and A2.0). The total mass of each component was determined from literature.^18,20,46^ The glucose mass requirement for each component was determined by utilizing microbial yields (g product/g glucose) and microbial titers (g/L of media) from literature sources (see Appendix B). Microbial yields with greater than 0.01 g product/g glucose were utilized (if available in literature), since the glucose concentration can vary depending on organism growth requirements, fermentation system, and operating parameters.^50^

When a microbial yield was unavailable for a growth medium component, microbial titers (g/L) from the literature were used to estimate the required mass of glucose. The glucose concentration of the medium was assumed to be 10 g/L for calculations, which utilized titer to estimate the required glucose mass. A batch system without the capabilities to add additional nutrients and glucose was assumed. Given this assumption, a glucose concentration of 10 g/L was deemed acceptable.^51^

The inputs/outputs other than glucose for microbially produced compounds were estimated by using industrial lysine production for proxy data. Varying yields between compounds indicated that a correction factor was necessary, i.e., more resources are utilized if more batches are required for the same mass of product. Each correction factor was calculated utilizing the reported lysine yield and the reported compound yields.^52^ When the microbial titer was reported and used in the model, an assumed glucose concentration (10 g/L) was used to calculate the correction factor. Tables A1 and A2 in Appendix B provide correction factors and sources for yields and titers (see calculations A2 and A3 in Appendix B).

##### Enzyme-Derived Products

The embedded resources for the enzymatic production of E8/B9 components were estimated utilizing a similar approach as previously described in the Microbial Fermentation Products section. l-Aspartic acid was the only E8/B9 component identified to be produced enzymatically, and the description of the assumed process can be found in Appendix C.

##### Chemical Products

The ecoinvent database was utilized to estimate embedded energy and material flows for compounds produced via the chemical synthesis.^47^ If the ecoinvent data sets were not available, reported production methods for the compounds were analyzed and stoichiometric calculations were conducted to determine the mass of E8/B9 component precursors (reactants). This process was repeated if the E8/B9 precursor was not available in the ecoinvent data set. The Supporting Information provides additional clarification for the stoichiometric calculation procedure. Substitution was also utilized if the data were unavailable in the ecoinvent data set for particular E8/B9 components (e.g., ascorbic acid was substituted for ascorbic acid 2-phosphate).

The material and energy flows for these compounds were tracked and aggregated by using the OpenLCA software. Table A3 in Appendix D provides a list of each component and the components’ precursors. If industrial production information was unavailable, embedded resources could not be quantified, or a reasonable substitute could not be identified, then no data were entered. Components without data were still entered into OpenLCA, but without any inputs or outputs. It should be noted that the described method for estimating the inputs and outputs should be considered nonexhaustive due to these gaps in the data.

##### Solvay and Potash

These categories of E8/B9 components utilize soda ash and potash as major components in their manufacture. For these components, both ecoinvent and available literature estimates were utilized in the same manner as previously described in the chemical category.

##### Brine Evaporation

Sodium chloride utilized as an E8/B9 component or for other component production processes is assumed to be produced from a mix of brine and mining operations. Sodium chloride in brine is utilized for soda ash production and is accounted for by utilizing the brine production data set, which does not include cleaning and drying steps. The reported embedded resources for nonsoda ash-related sodium chloride production include extraction, drying, and purification.

##### Animal Cell-Produced Product

TGF-β can be produced using animal cell culture (Beatson et al., 2011; Zou & Sun, 2004). One advantage of producing TGF-β via animal cell culture rather than a more traditional fermentation organism like *Escherichia coli* is the absence of endotoxin. One disadvantage is that the growth medium must be suitable for animal cell culture, which has more complex nutrient requirements. This analysis assumes that TGF-β was produced via animal cell culture. Chinese hamster ovary (CHO) cells are the most used animal cell line and are particularly important for glycoprotein overexpression^53,54^ CHO cells require a more complex growth medium as compared to more basic media inputs used for bacteria or yeast growth. DMEM/F12 was utilized as the basal medium for E8 and B9 and was deemed to be an acceptable growth medium for CHO cells. The CHO cells were assumed to not require the other seven components of E8/B9 (ascorbic acid 2-phosphate, additional NaHCO_2_, sodium selenite, insulin, transferrin, and FGF-2).^18^ The material and energy flows were estimated for TGF-β using the data collected for the basal medium production and reported titers of TGF-β.

##### E8/B9 Components and Precursors Utilized in Multiple Production Processes

Several E8/B9 component precursors are used in the production of multiple E8/B9 components. The material and energy flows necessary to produce these components were accounted for utilizing ecoinvent data sets.^47^Appendix G lists the components that are utilized in the production of multiple E8/B9 components.

##### Components Not Included in the Assessment

Lipoic acid and HEPES are not accounted for due to the authors’ inability to find either production or environmental impact data. Additional information about the production of these components can be found in Appendix H.

##### Additional B9 Components

The composition of B9 is similar to E8, but has additional components: neuregulin, antibiotics/antimycotic, ultrapure water, and recombinant albumin. Additional analysis was conducted to evaluate the environmental impacts of these supplemental components. Antibiotic/antimycotic production typically utilizes 100 kg of solvent and 50 kg of water per kilogram of compound produced.^55^ An ecoinvent-provided equal mix of 15 different organic solvents (acetone, butanol, cumene, cyclohexanol, dichloromethane, ethylbenzene, ethyl glycol, isopropanol, methanol, methyl ethyl ketone, nitrobenzene, styrene, tetrachloroethylene, toluene, and xylene) was utilized to estimate the impact of generic organic solvent use. The neuregulin and recombinant albumin environmental impacts were estimated utilizing reported titers (5 mg/L and 17 g/L, respectively) and the method described in microbial titer methods section.^56,57^

##### Cultured Meat Process Model

**Figure 1:**
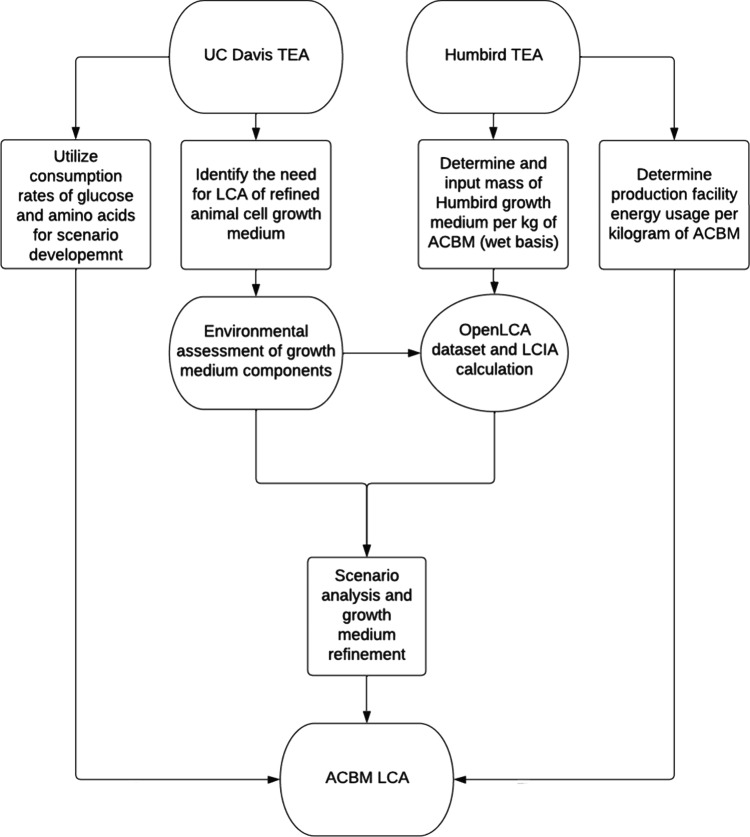
Workflow diagram for creating a process model and LCA from previous TEA studies.

The development of a process model is an important element in identifying the inputs and outputs of a system. The Risner et al. and Humbird TEAs are the most complete studies that contribute to our understanding of the cultured meat production process currently. This study seeks to leverage the best components of both TEA models to inform the design of the cultured meat process model for our LCA. Figure 1 highlights how each study contributed to the overall design of this LCA.

Both TEAs highlight the importance of the growth medium in influencing the economic viability of future cultured meat products, and this parameter serves as an important parameter for defining the scenarios for our analysis.

The Risner et al. TEA estimated the required volume of growth medium based on cellular glucose consumption rates and did not examine cellular amino acid consumption rates at the time. However, animal cells must have an amino acid source. The theoretical limit of the mass balance of the amino acids provided and the protein produced is 1:1. In reality, it is lower since amino acids are also used as an energy source as well as for nucleic acid production. In this study, cells were assumed to have a dry matter content of 30%, which consists of 70% protein, 15% lipids, 10% carbohydrates, and 5% nucleic acid.^9^

**Figure 2:**
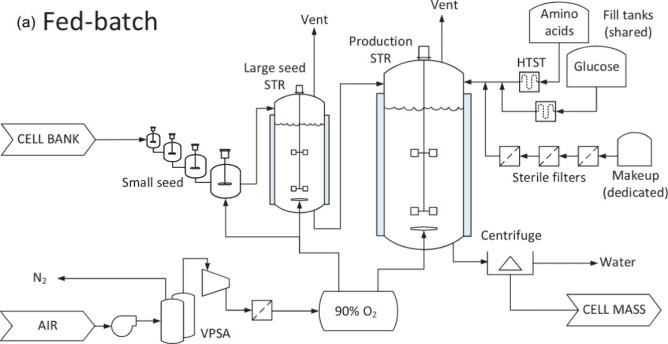
Fed-batch cultured meat production system utilized in this LCA of cultured meat. This image was taken from scale-up economics for cultured meat.^9^

These are key assumptions for the new model, which explores utilizing both the minimum glucose and amino acid requirements to generate minimum viability scenarios for our production system. We have taken the approach of utilizing a fed-batch system that supplies the cells with the nutrients in E8 as necessary (Figure 2). This approach allows for a concentrated feed to be added to the bioreactors and prevents cells from experiencing issues related to osmotic pressures from increased nutrient concentrations. “Scenario 1—Glucose consumption rate” utilizes a glucose requirement of 1148 L of E8 to produce a kilogram of cultured meat. “Scenario 2—Amino acid requirement” scales E8 provision to match the amino acid requirements for cell cultivation, requiring ∼292 L of E8 to produce a kilogram of cultured meat.

To determine the cellular metabolic requirements, Humbird examined a “wild-type” cellular metabolism and an “enhanced” cellular metabolism was examined. The wild-type metabolism was deemed too inefficient for economic production due to lactate and ammonia production, which inhibit cell growth. Thus, we only included the enhanced cellular metabolism, which is approximately twice as efficient as the wild-type metabolism in our analysis. Equation 1 was utilized in the Humbird TEA to determine the mass of glucose, oxygen, and amino acids needed for cellular proliferation. Dry cell matter (DCM) was determined, and the mass of each compound needed to produce a kg of cultured meat (wet basis) was calculated.

Enhanced cellular metabolism from Humbird TEA
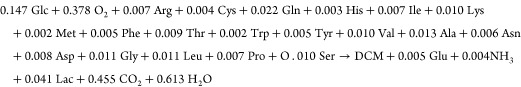
1

Humbird’s cellular metabolism model assumes the use of glutamine, but glutamine is not an E8 component. To address this gap, microbial yield (0.368 g/g glucose) was identified in the literature and input to the microbial method to determine its environmental impact as a component of the growth medium.^58^ While glutamine as an input is challenging due to stability issues, we include it in our assessment as it plays an important role in cellular metabolism.^59^ Masses of minor protein ingredients such as insulin, transferrin, FGF, and TGF were also accounted for on a functional unit basis.

The Humbird TEA also accounted for the power consumption per batch. We examined the energy usage based upon batches per year (54,000 batches per year at 1852 kg/batch). Supporting Information provides energy usage and unit conversions. This was then examined on the basis of a functional unit of 1 kg of cultured meat (∼33 MJ/kg of ACBM).

In summary, our integrated process model utilized the more complete accounting of energy use and capital expenditures from the Humbird TEA, and the more thorough assumptions about the growth medium (including additional necessary vitamins and minerals for animal cell growth) from the Risner et al. TEA.

Utilizing this new integrated process model and environmental data from the growth medium components, a new production system was modeled to understand the near-term environmental impact of cultured meat production. Our LCA focused on operational inputs and did not include assessment of the large capital assets for cultured meat production, e.g., bioreactor construction. The energy requirements from the production facility modeled from Humbird were used to estimate the energy inputs. Finally, the growth medium requirements described above were entered into OpenLCA to link each of these inputs to the environmental input data sets for the growth medium components.

### Life Cycle Impact Assessment

After all the inputs were identified and consolidated, a life cycle impact assessment was completed utilizing data and methods from the environmental assessment of the growth medium components, OpenLCA v.1.10 software, and OpenLCA LCIA v2.1.2 methods software. The Tool for Reduction and Assessment of Chemicals and other Environmental Impacts (TRACI) 2.1 was the LCIA method utilized in the OpenLCA software, and these results were combined with the facility power data to estimate the total potential environmental impact of the production of 1 kg of cultured meat (wet basis).

#### Scenario Analysis

All scenarios utilize a fed-batch system as described in the Humbird TEA. Energy estimates from the Humbird TEA are utilized in all scenarios. Growth medium components were assumed to be delivered to the animal cells as needed, and the buildup of growth inhibiting metabolites such as lactate or ammonia is not accounted for unless specifically stated in the scenario. The growth medium substrates are also assumed to be supplied via a fed batch to achieve the highest possible specific growth rate in the production bioreactor. The three main scenarios were defined by utilizing data from the Risner et al. and Humbird TEAs. Detailed descriptions for each of the scenarios are provided below:

1. Scenario 1—glucose consumption rate (GCR): Reported estimates of the cellular GCR were utilized to estimate the required growth medium volume in the Risner et al. TEA. This is the same nutrient requirement as scenario 1 from the Risner et al. TEA; however, it is being delivered in a fed-batch manner as described by the Humbird system. The entire volume of growth medium is not assumed to be replaced, but the required nutrients are added as needed. This scenario utilizes E8 for its growth medium, and it is estimated to require the equivalent of 1148 L of E8 to produce 1 kg of cultured meat wet basis.
2. Scenario 2—amino acid requirement (AAR): This scenario utilizes E8 as its growth medium and provides the minimum amount of amino acids needed to achieve the minimum amount of cellular protein mass for 1 kg of cultured meat to be produced. This scenario indicates that 291.5 L of E8 would contain the necessary amount of amino acids to produce a kilogram of cultured meat wet basis with 21% (w/w) protein content.
3. Scenario 3—enhanced Humbird growth medium (HGM): This scenario utilizes the Humbird TEA enhanced metabolism equation (eq 1) to estimate the total required growth medium nutrients. This scenario utilizes 0.35 kg of glucose, 0.16 kg of oxygen, 0.26 kg of amino acids, and minor protein ingredients (209.52 mg of insulin, 115.56 mg of transferrin, 1.08 mg of FGF and 0.02 mg of TGF) to produce 1 kg of cultured meat wet basis.

While these main scenarios represent the core of our LCA, they all assume that the main ingredients of E8 are produced using food- or feed-grade production systems. Given the fact that cultured meat production is originally based on pharmaceutical systems for producing monoclonal antibodies, it is a significant assumption that cultured meat can even be produced using food/feed-grade inputs. Currently, in animal cell culture, growth mediums are highly refined to prevent contamination.^60^

#### Purification Factor

A critical component to our approach in this study was differentiating the supply chain inputs between pharmaceutical grade and commodity inputs. Given that the ingredient supply chain for ACBM does not exist yet at commodity scale, an established “purification factor” was estimated by leveraging previous studies on bulk chemical vs pharmaceutical chemical production. In these previous studies, it was determined that pharmaceutical chemical production is more energy and resource intensive than bulk chemical production with the cumulative energy demand (MJ) 20× greater than bulk chemical production and the global warming potential (GWP) 25× greater than bulk chemical production.^43^ It has also been illustrated that the production of recombinant proteins utilized in animal cell culture such as IGF-1, FGF, and TGF-β has significant global warming potential (0.1, 0.04, and 0.2 kg CO_2_ eq per milligram, respectively).^61^ Given a lack of individual data on pharmaceutical ingredients, we utilized an overarching purification factor (PF) of 20× was utilized to reflect the level of refinement used for laboratory or pharmaceutical-grade animal cell culture components. We applied this PF to each of the three base scenarios to estimate pharmaceutical-based scenarios, and thus generating a total of six scenarios in the assessment.^43^

## Results

Initially, we conducted an LCA of E8 and B9 to understand how animal cell growth medium choice could potentially influence the environmental impact of animal cell culturing. The data and results from the E8/B9 LCA were then utilized to inform the LCA of cultured meat across the previously described production scenarios. After each initial scenario was examined, a purification factor was applied to each scenario to provide an estimate of the environmental impact of cultured meat if pharmaceutical-grade growth medium components are utilized for production, as described in the methods.

We examined the environmental impact of 1 L of both E8 and B9 growth media. The baseline results indicate a dramatic difference in E8 and B9, mostly due to the inclusion of antibiotics in the B9 formulation. When an antibiotic-free version of B9 (B9af) is considered, the energy use and environmental impacts are analogous to those of E8. The LCIA results for TRACI LCIA methods for the E8, B9, and B9af are shown in Table 1.^24,45−47^

**Table 1:** TRACI Impact Category Results for 1 L of Growth Medium.^a^

	DMEM/F12 basal media	Essential 8	Beefy-9 no antibiotic	Beefy-9
smog (kg O_3_ eq)	3.66 × 10^–03^	3.89 × 10^–03^	3.73 × 10^–03^	4.06 × 10^–01^
acidification (kg SO_2_ eq)	5.30 × 10^–04^	5.60 × 10^–04^	5.20 × 10^–04^	3.43 × 10^–02^
respiratory effects (kg PM2.5 equiv)	6.62 × 10^–05^	7.05 × 10^–05^	6.65 × 10^–05^	4.65 × 10^–03^
non carcinogenic (CTUh)	–1.62 × 10^–08^	–1.56 × 10^–08^	–1.36 × 10^–08^	1.08 × 10^–06^
ecotoxicity (CTUe)	1.50 × 10^00^	1.61 × 10^00^	1.50 × 10^00^	6.15 × 10^01^
global warming potential (kg CO2-eq)	6.20 × 10^–02^	6.57 × 10^–02^	6.40 × 10^–02^	8.03 × 10^00^
ozone depletion (kg CFC-11 equiv)	5.75 × 10^–09^	6.00 × 10^–09^	7.11 × 10^–09^	2.92 × 10^–05^
carcinogenics (CTU)	7.09 × 10^–09^	7.55 × 10^–09^	7.07 × 10^–09^	3.65 × 10^–07^
eutrophication (kg N eq)	3.80 × 10^–04^	3.90 × 10^–04^	3.90 × 10^–04^	1.18 × 10^–02^
fossil fuel depletion (MJ surplus)	7.10 × 10^–02^	7.43 × 10^–02^	7.70 × 10^–02^	3.33 × 10^01^

a Levels of noncarcinogenic ecotoxicity reported as near zero negative and positive values according to LCIA software. PM2.5, particles less than 2.5 μm in diameter; CTUh, comparative toxic unit for humans; CTUh per kg emitted = disease cases per kg emitted; CTUe, comparative toxic unit for aquatic ecotoxicity impacts; CTUe per kg emitted = PAF × m^3^ × day per kg emitted; PAF, potentially affected fraction of species; CTU, comparative toxic unit

The results of the LCIA indicate minimal differences in DMEM/F12 basal media, E8, and B9af growth mediums (Table 1). When antibiotic containing growth mediums are included, the B9 LCIA results are orders of magnitude higher than those of E8 and DMEM/F12 growth mediums across most impact categories. Thus, from an environmental perspective, the reduction or elimination of antibiotic/antimycotic growth medium components would be particularly advantageous. If antibiotics/antimycotics are utilized in the ACBM manufacture, this would also pose an additional food safety risk.^62^ It is also important to note that this analysis does not account for antibiotics being released into the environment during production.

**Figure 3:**
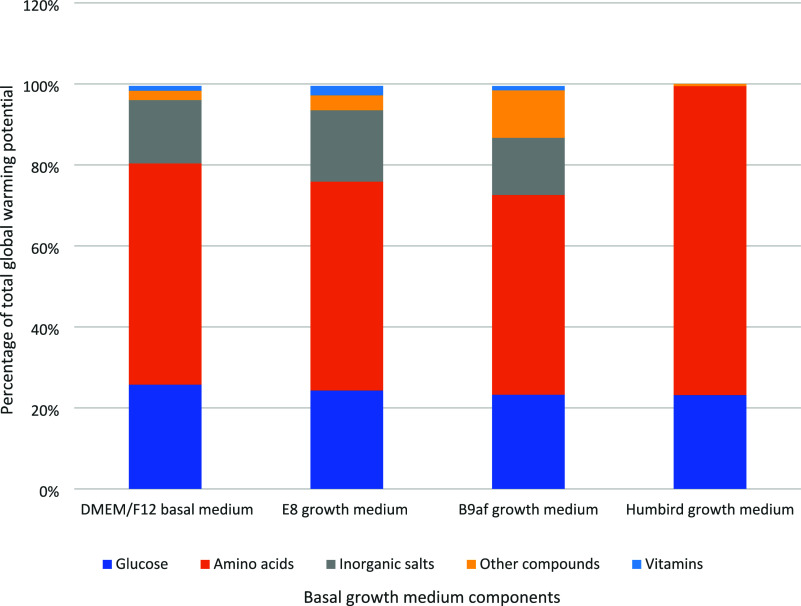
Growth medium component contribution to global warming potential of each basal growth medium.

The LCA results suggest that the DMEM/F12 basal media component of the E8 and B9af growth mediums is responsible for the majority of the environmental impacts (>90%) of each medium. Figure 3 compares the global warming potential of the different categories of basal growth medium components within each growth medium and illustrates differences in the basal mediums, such as the inclusion of vitamins, inorganic salts, and other components in the various growth mediums. For all four growth media, the total mass of amino acids had the greatest influence on the global warming potential of the growth mediums. To further analyze the environmental impacts of DMEM/F12, a sensitivity analysis was conducted on each DMEM/F12 component. This analysis found that glucose was the most environmentally impactful component of the DMEM/F12 medium, and this is largely due to its relatively high concentration (3.151g/L) in relation to the other DMEM/F12 growth medium components. However, the environmental impact of the HEPES buffering agent (3.575 g/L) could not be accounted for due to the authors’ inability to find environmental data related to its production process.

After the comparison of growth mediums, an LCIA was conducted on the three base scenarios for producing 1 kg of cultured meat as well as these same scenarios modified by applying a purification factor to reflect the influence of utilizing pharmaceutical-grade inputs instead of food- or feed-grade inputs. The results for all six scenarios are summarized in Table 2. As B9 (and B9af) is not commonly used in ACBM production, we did not include it as a process input for our complete cultured meat LCA, electing to analyze only E8 and Humbird’s growth media in our analysis to simplify results.^18,20,44,45^

**Table 2:** TRACI 2.1 LCIA Results for Each Unprocessed and Purified Growth Medium Scenarios

	GCR	GCR-PF	AAR	AAR-PF	HGM	HGM-PF
smog (kg O3 equiv)	4.5	89.4	1.1	22.7	0.69	13.8
acidification (kg SO2 equiv)	0.6	12.9	0.2	3.3	0.10	1.9
respiratory effects (kg PM2.5 equiv)	0.1	1.6	0.0	0.4	0.01	0.3
non carcinogenic (CTUh)	0.0	0.0	0.0	0.0	0.00	0.0
ecotoxicity (CTUe)	1848.9	36,977.9	469.6	9391.7	229.92	4598.4
global warming potential (kg CO2 equiv)	75.4	1508.3	19.2	383.1	12.31	246.1
ozone depletion (kg CFC-11 equiv)	0.0	0.0	0.0	0.0	0.00	0.0
carcinogenics (CTU)	0.0	0.0	0.0	0.0	0.00	0.0
eutrophication (kg N eq)	0.5	9.0	0.1	2.3	0.07	1.4
fossil fuel depletion (MJ surplus)^a^	85.3	1706.4	21.7	433.4	14.89	297.8

a Energy usage by cultured meat production facility not accounted for in the table. GCR, glucose consumption rate scenario; AAR, amino acid requirement scenario; HGM, enhanced Humbird growth medium scenario; PF, purification factor.

**Figure 4:**
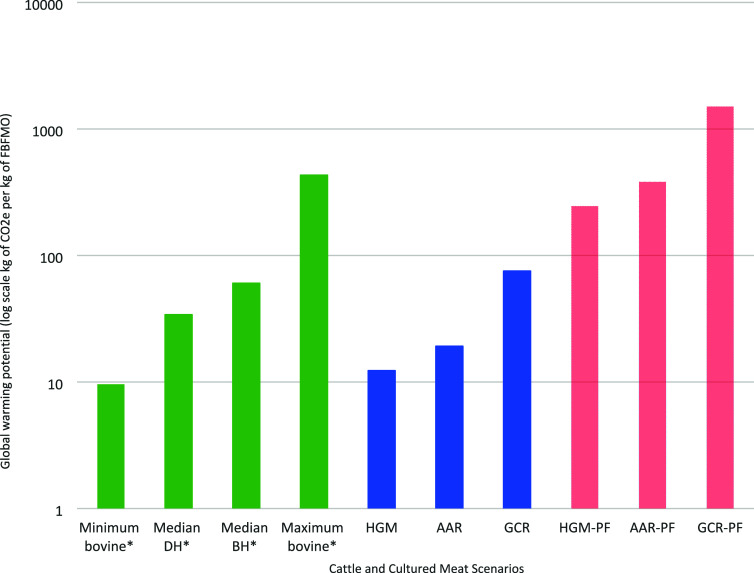
Log-scale GWP comparison of the six cultured meat production scenarios (three process models each with and without purification factor applied) relative to reported retail beef values (FBFMO). DH, dairy herd; BH, beef herd; GCR, glucose consumption rate scenario; AAR, amino acid requirement scenario; HGM, enhanced Humbird growth medium scenario; PF, purification factor. Reported retail beef from Reducing food’s environmental impacts through producers and consumers.^11^

The GWP for all cultured meat scenarios (ranging from 12 to 1508 kg of CO_2_e per kilogram of cultured meat) ranged from ∼80% less than to ∼2513% more than the median GWP of retail beef, but all were greater than the minimum reported GWP for retail beef (9.6 kg of CO_2_e per kg of FBFMO).^11^ The GWP of all purified scenarios ranged from 246 to 1508 kg of CO_2_e per kilogram of cultured meat, which is 4 to 25 times greater than the median GWP of retail beef (∼60 kg CO_2_e per kg of FFBMO).^11^Figure 4 illustrates the difference in the GWP of retail beef and cradle to upstream of cultured meat production gate.

To understand the role of cattle feed to beef production, a recent LCA concluded that the production of total mix ration (TMR) for finishing beef in a feedlot contributed 0.521–0.63 kg CO_2_e/kg TMR.^63^ These rations, which were consumed at rate of 1900–4495 kg per head of cattle, were reported to contribute ∼4–12 kg of CO_2_e per kg of primal beef cuts (∼7–20% of the reported median GWP for beef) from a ∼635 kg animal,^63^ whereas the animal cell growth medium (i.e., the feed for the cells) was responsible for nearly the entirety of this study’s reported GWP.

**Figure 5:**
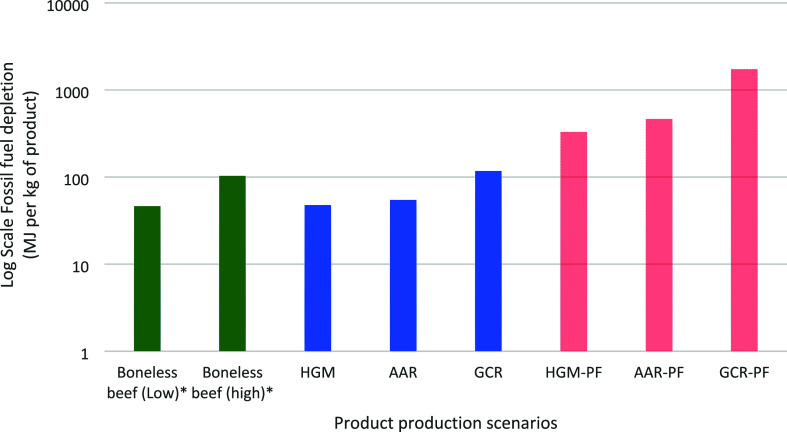
Log-scale fossil fuel depletion comparison of the six cultured meat production scenarios (three process models each with and without purification factor applied) of each cultured meat production scenario in comparison with boneless beef. *These are energy intensities which may include nonfossil fuel energy.^64^ GCR, glucose consumption rate scenario; AAR, amino acid requirement scenario; HGM, enhanced Humbird Growth Medium scenario; PF, purification factor.

The fossil fuel depletion metrics were greater for all of the cultured meat production scenarios as compared to the low boneless beef metric (Figure 5). For unpurified scenarios, the higher level of energy use is largely associated with upstream processing facilities producing input products required for cultured meat production. The HGM scenario was approximately ∼1 MJ per kilogram greater than the lower estimate for boneless beef.^64^ The AAR-PF and AGM-PF scenarios with growth mediums refined for animal cell culture required approximately an order of magnitude more energy than the reported low for boneless beef. The high cumulative energy demand for boneless beef was approximately double the fossil fuel depletion of AAR and HGM scenarios. The fossil fuel depletion for scenarios with purified growth medium components were approximately 3 to 17 times greater than the reported high for boneless beef.

Our system boundary for cultured meat production does not include postharvest handling, storage, and transport, which all require energy in some form. Many of these postproduction processes are included in the reported GWP estimates for retail beef; however, a reported mean GWP for these postproduction processes is less than 1 kg CO_2_e per kilogram of meat.^11,65^

## Discussion

Our results indicate that cultured meat is not necessarily a less resource-intensive protein product than conventional meat and, in fact, may lead to significantly greater environmental impact if the industry is unable to fully transition from pharmaceutical-grade ingredients to food/feed-grade inputs. This transition represents a significant challenge, given that growth media will likely need to be optimized for individual cell lines to achieve cell densities beyond current pharmaceutical industry performance. It should also be noted that these results should be considered a minimum since the environmental assessment of the growth medium components is admittedly nonexhaustive.

In this evaluation, our primary focus has been on the resource intensity of the growth media. We have largely focused on the key growth medium components (e.g., glucose, amino acids, vitamins, growth factors, salts, and minerals) and acknowledge the uncertainty given the quantity and complexity of these calculations. That said, the core scenario analysis (i.e., no purification factor) should be viewed as minimum environmental impacts due to several factors, including incomplete data sets, the assumption of the broad growth of a bioeconomy to supply ACBM inputs (e.g., amino acids, among others), and the exclusion of energy and materials required to scale the cultured meat industry.^61^

One example of having incomplete data is that none of the data sets utilized in this LCA accounted for the purification of growth medium components for laboratory and pharmaceutical animal cell culture. Due to the lack of data related to this, we utilized a purification factor based on the comparison of fine chemical and bulk chemical production.^43^ Additionally, this study does not account for HEPES or lipoic acid production, and there is only partial accounting of the embedded resources and energy for other E8 components (see Supporting Information). Furthermore, many of these growth medium components were assumed to be produced microbially via a large, concomitant bioeconomy supply chain that develops in parallel to ACBM production systems.

In our analysis, we assessed the environmental impact per unit of ACBM produced but did not consider the total environmental impact of scaling up cultured meat production facilities into a mature food industry. In 2021, the total cell culture bioprocessing capacity was 17,400,000 L with mammalian cell culture capacity being 11,750,000 L.^66^ Based on the Humbird TEA, to achieve 1% of current global meat production (∼3-million metric tons), each fed-batch production facility would require a total bioreactor volume of 649,000 L and that it would require ∼440 identical facilities, or an additional 300,000,000 L of mammalian cell culture capacity, representing an ∼3000% increase in capacity. If this capital expansion was included in our LCA, we would need to expand our system boundary to include all the input energy and materials for the construction of these facilities. We also have not included the environmental impacts associated with scaling up multiple production facilities to produce the required mass of growth media components necessary for cultured meat production at scale.^9,36^ Expanding the system boundary to include this level of scaling would inherently increase the environmental impact of cultured meat production.

As a result of a highly expanded future bioeconomy, we assumed significantly improved production efficiencies. Scenarios AAR and AAR-PF assume a 100% conversion of amino acids to protein. This assumption is probably not achievable under even the best fermentation conditions given that the amino acids also supply the nitrogen atom and amino group in the synthesis of nucleotide bases and nitrogen-containing sugars.^67^ The amino acid carbon skeleton is also utilized in the formation of groups like the functional methyl group.^67^ For example, the most optimized ethanol fermentations are unable to achieve theoretical yields due to carbon being utilized to produce other metabolites such as glycerol.^68−70^ This indicates that AAR-PF may be an unlikely minimum, as well. This study also assumes that growth factors are produced in a manner similar to that for industrially scaled amino acid production, and this is currently not the case.

Our analysis also does not include a detailed assessment of the production of growth factors, which play an important role in animal cell culture. Growth factors are utilized for the development of a serum-free growth medium for animal cell culture with the idea of replacing key signaling compounds in serums such as FBS. A recent study suggests that growth factor production will likely have a substantial environmental impact (0.1 kg CO_2_ eq per milligram of IGF-1, 0.04 kg CO_2_ eq per milligram of FGF, and 0.2 kg CO_2_ eq per milligram of TGF-β).^61^ Including the reported growth factor mass utilized to produce a kg of cultured meat in the Humbird growth medium (209 mg of insulin, 115 mg of transferrin, 1 mg of FGF, and 0.02 mg of TGF per kg of cultured meat) would increase GWP by ∼21 kg of CO_2_e without including transferrin production.

The scenarios utilizing our relatively crude multiplication factor should be carefully considered. Large-scale plant contamination has been experienced at biopharmaceutical facilities, and one such instance caused a revenue loss of 100–300 million USD.^71^ Utilizing less-refined growth medium components would likely increase the risk of contamination for cultured meat production, potentially causing a facility to undergo resource-intensive decontamination processes. The economic risk of contamination is currently illustrated by the industrial shift to single-use bioreactors for monoclonal antibody production to reduce costs associated with contamination.^72^

In addition, a more refined growth medium would likely be required to achieve advances in cell line optimization. Utilizing less-refined growth medium components would increase the risk for cell exposure to contaminants and inhibit the ability of the cells to proliferate to cell densities greater than those of current biopharmaceutical standards. Animal cell culture is inherently different than culturing bacteria or yeast cells due to their enhanced sensitivity to environmental factors as well as chemical and microbial contamination. Contamination from a variety of substances including typical contaminants such as bacteria, mycoplasma, viruses, and endotoxin can cause a variety of issues (e.g., resource competition, cell death) within animal cell cultures.^73^ Viral contamination is a high risk for serum,^73^ and viral filtration would be likely necessary for operation if heat-sensitive growth medium components are utilized. The use of viral filters would further increase resource use estimates, as this process was outside the scope of our model. Even contaminants such as plasticizers and trace elements can affect cell culture.^73^

Even with the data gaps and model uncertainty already discussed, the scenario results from our model should be carefully considered by all ACBM stakeholders. To counteract some uncertainty, the authors chose to utilize a scenario analysis, which examines the growth medium from a food/feed- and pharmaceutical-grade perspective. For these reasons, we believe that additional work is necessary to provide this expanded view of the environmental impact of producing cultured meat at scale. As more information and data become available, more comprehensive analyses should be conducted.

Critical assessment of the environmental impact of emerging technologies is a relatively new concept, but it is highly important when significant changes to societal-level production systems are proposed.^74^ Agricultural and food production systems are central to feeding a growing global population, and the development of technology that enhances food production is important for societal progress. Evaluation of these potentially disruptive technologies from a systems-level perspective is essential for those seeking to transform our food system. Ideally, a robust environmental assessment of novel food technologies will allow policymakers and investors to make informed decisions on the allocation of capital.

While cultured meat has been proposed as a technological solution to meet the growing global demand for protein without placing undue burden on the planet, this analysis suggests that cultured meat production is not inherently environmentally friendly but rather carries a significant risk of having a greater environmental impact than conventional meat production. These results are contrary to many of the existing LCAs of cultured meat because their technology models are generally based on significant assumptions in technological advancement, while the goal of our study was to accurately reflect the most detailed current and near-term process systems in this emerging food technology sector. We acknowledge that significant technological advancement in processing is likely to take place and, in fact, needs to take place for cultured meat to be economically competitive. However, we argue that until these emerging approaches are proven and adopted at scale, we need to understand the environmental impact of current systems to provide a baseline understanding of industry practice. In this way, we hope to highlight that achieving environmental benefits needs to be a design criterion for technology advancement and not assumed to be an inherent outcome of the product itself. This is an important conclusion given that investment dollars have specifically been allocated to this sector with the thesis that this product will necessarily be more environmentally friendly than beef and other conventional meat products.

To realize environmental benefits via scaled production of this product will require resolving key challenges. The first and most important challenge will be developing a highly optimized, environmentally friendly growth medium that allows for the proliferation of animal cells at high cell densities. Additionally, we will need bioreactors that are larger than the proven scale and which utilize aseptic systems with viral exclusion. Perhaps a focus on advancing these precompetitive scientific advances will lead to a better outcome for all.

In summary, discerning the minimal environmental footprint of emerging cultured meat technologies is vital for policymakers and investors committed to fostering initiatives with dual economic and environmental returns. Our findings indicate that cultured meat may not outperform traditional meat production in environmental terms, especially as our foundational model likely underestimates this impact due to the nascent stage of the industry and therefore assumptions made throughout our LCA. We have strived for transparency in our LCA to facilitate stakeholders’ understanding of our rationale and the derived conclusions. More generally, this research underscores the necessity of integrating comprehensive TEAs with LCAs to accurately evaluate the environmental consequences of the development of novel food and agricultural technologies.

## Supporting Information Available

Essential 8 and Beefy-9 growth medium composition, phenol red example for ecoinvent dataset utilization, Humbird TEA energy estimates, raw ingredients, microbial yield, reported microbial titer for some E8/B9 components (PDF)

## Abbreviations

AA: Amino acids
AAR: Amino acid requirement
ACBM: Animal cell-based meat
B9: Beefy-9 growth medium
B9af: Antibiotic-free Beefy-9 growth medium
BH: Beef herd
CD: Chemically defined
CHO: Chinese hamster ovaries
CO2e: Carbon dioxide equivalent
CTU: Comparative toxic unit
CTUe: Comparative toxic unit for aquatic ecotoxicity impacts
CTUh: Comparative toxic unit for humans
DCM: Dry cell matter
DH: Dairy herd
DMEM/F12: Dulbecco’s modified Eagle medium/Hams’ F12 (DMEM/F12) basal medium
E8: Essential 8^TM^ growth medium
FBFMO: Fat- and bone-free meat and edible offal
FBS: Fetal bovine serum
FGF-2: Fibroblast growth factor
GCR: Glucose consumption rate
GHG: Greenhouse gas
GWP: Global warming potential
HEPES: 4-(2-Hydroxyethyl)-1-piperazineethanesulfonic acid
HGM: Humbird growth medium
IGF-1: Insulin-like growth factor
ISO: International Organization for Standardization
LCA: Life cycle assessment
LCI: Life cycle inventory
LCIA: Life cycle impact assessment
PAF: Potentially affected fraction of species
PF: Protein free
PF: Purification factor
PM2.5: Particles less than 2.5 μm in diameter
TEA: Technoeconomic assessment
TGF-β: Transforming growth factor beta
TMR: Total mix ration
TRACI: Tool for reduction and assessment of chemicals and other environmental impacts
XP: Brand of animal cell growth medium
