# Supplementary material for: Environmental Impacts of Cultured Meat: A Cradle-to-Gate Life Cycle Assessment
Source: ACS Food Sci Technol. 2024 Dec 30;5(1):61–74. doi: 10.1021/acsfoodscitech.4c00281 (PMC11744764; doi:10.1021/acsfoodscitech.4c00281)
Supplement: Supplementary file 1 — fs4c00281_si_001.pdf [file fs4c00281_si_001.pdf]

Title: Environmental impacts of cultured meat: A cradle-to-gate life cycle assessment

Authors: Derrick Risner, Patrick Negulescu, Yoonbin Kim, Cuong Nguyen, Justin B. Siegel and Edward S. Spang

Summary: The twenty additional pages, 8 tables and 3 figures provide additional details on how the results were determined via calculation and assumptions. The appendix sections cover the assumptions and calculations related to resource use for growth medium production.

Supplemental tables and figures.

Table 1.0 Essential 8 and Beefy-9 growth medium composition

**Component**	**Concentration in Essential 8^{TM}** (µg/mL)	**Concentration in Beefy-9** (µg/mL)
DMEM/F12 basal media	-	-
2-Phospho-L-ascorbic acid trisodium salt	64	200
Insulin (human, recombinant)	19.4	20
Transferrin (human, recombinant)	10.7	20
Sodium selenite	0.014	0.02
Fibroblast growth factor (FGF-2)	0.1	0.04
Neuregulin (NRG1)	-	0.0001
Transforming growth factor (TGF-β3)	0.002	0.0001
UltraPure Water	-	58000
Antibiotic/Antimycotic	-	10000
Recombinant albumin	-	800
Additional $NaHCO_3$	543	-

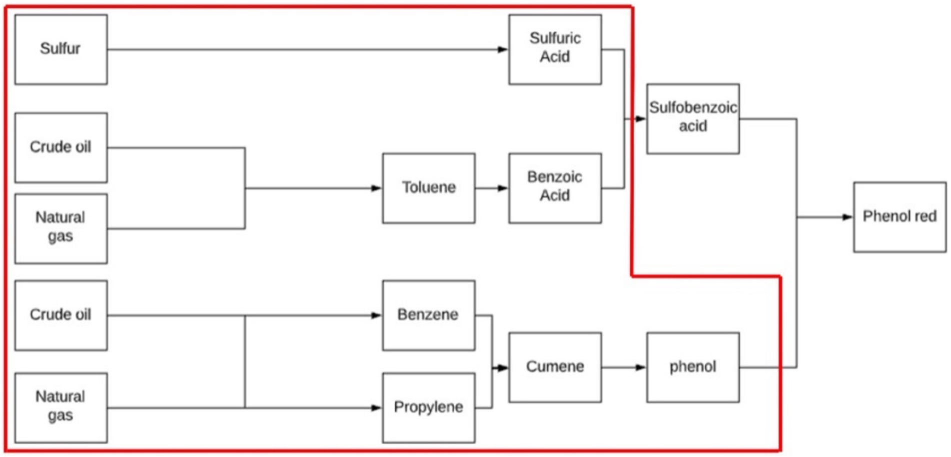

Figure 1.0 Phenol Red example for ecoinvent dataset utilization.
Compounds within the red outline have ecoinvent datasets which account for their material and energy flows. The material and energy flows are not accounted for components outside the red outline. Stoichiometric calculations (theoretical yield utilized) were conducted to estimate mass of each compound when information was not available in ecoinvent (Ex. A minimum of 0.519 kg of sulfobenzoic acid and 0.5311 kg of phenol is needed to produce 1 kg of phenol red).

Figure 1.0 provides an example of this process with the components encased in the red outline having datasets available in ecoinvent and these datasets are utilized to account for the environmental impact of each component.

Table 2. Humbird TEA energy estimates

Unit Operation	kWh per batch	BTU/year	MJ/year
Vacuum pressure swing adsorption oxygen gas generator power	2,139	394,106,472,000	415,805,974
Compressor power	156	28,742,688,000	30,325,260
Agitator	47	8,659,656,000	9,136,457
Chiller	257	47,351,736,000	49,958,923
Dewatering	22	4,053,456,000	4,276,639
Facility*	11,511	2,120,878,728,000	2,237,654,311
Natural gas (reported in MMBTU)	N/A	540,000,000,000	569,732,400
Total	14,132	3,143,792,736,000	3,316,889,964

*Includes clean room facilities

Appendix A- Raw ingredients

Glucose
The material flow for glucose production was utilizing ecoinvent 3.8 datasets. The maize was assumed to be dried during initial harvest and flow the can be described in calculation A1. The conversion factors were all taken from the ecoinvent 3.8 datasets. Transportation was assumed between each step and is accounted for utilizing ecoinvents datasets.

Calculation A1. Example of ecoinvent mass determination: Maize mass needed for glucose production

Maize → Starch → Glucose

1 kg of glucose x 0.9 kg starch/1 kg glucose = 0.9 kg of starch
0.9 kg of starch x 1.261 kg of dry corn/1 kg of starch = 1.1349 kg of dry corn

Calculation notes:
1 kg of glucose requires 0.9 kg of starch to produce [1]
1 kg of starch requires 1.261 kg of maize [1]

Linoleic acid
Cottonseed oil production was utilized to estimate the linoleic acid production. The separation of linoleic acid from fatty acids was not accounted for and would likely increase the input/outputs of this E8/B9 component. The ecoinvent datasets were utilized to estimate the material/energy flow from cottonseed production to cottonseed oil refining. Transportation was assumed between each production step. Refined cottonseed oil was assumed to have a similar triglyceride content (96% w/w) [2]. Cottonseed oil's fatty acid profile has been reported consist of the 52% linoleic acid. This indicates that a minimum of ~2 kg of refined cottonseed oil would be required to produce 1 kg of linoleic acid.

Appendix B- Microbial yield

Table A1. Reported microbial yields and calculated correction factor for some E8/B9 components

Compound	Yield (g/g $_{glucose}$)	Correction factor	Source
i-Inositol [1]	0.820	0.788	[3]
L-Alanine	0.520	1.24	[4]
L-Arginine-HCl [2]	0.431	1.50	[5]
L-Asparagine-H_2O [2]	0.836	0.773	[4]
L-Cysteine-HCl-H_20 [2]	0.060	10.8	[6]
L-Cystine [2]	0.060	10.8	[6]
L-Glutamic acid	0.630	1.03	[7]
L-Histidine-HCl-H_2O [1,2]	0.080	8.08	[8]
L-Isoleucine	0.300	2.15	[4]
L-Leucine	0.150	4.31	[4]
L-Lysine-HCl [2]	0.646	1.00	[9]
L-Methionine[1]	0.130	4.97	[10]
L-Phenylalanine	0.180	3.59	[4]
L-Proline	0.360	1.79	[11]
L-Serine	0.430	1.50	[12]
L-Threonine	0.400	1.62	[4]
L-Tryosine	0.100	6.46	[13]
L-Tryptophan	0.200	3.23	[4]
L-Valine	0.280	2.31	[4]
Pyridoxal-HCl [2]	0.325	1.99	[14]
Pyridoxine-HCl [2]	0.325	1.99	[14]
Sodium Pyruvate [2]	0.678	0.953	[15,16]
Thymidine	0.019	34.0	[17]

[1]Coverted from mol/mol $_{glucose}$ to g/g $_{glucose}$
[2]See information below for additional clarification

Calculation A2. Correction factor calculation example for compounds with reported yields

L-Leucine correction factor = $0.646^{*} / 0.150^{**} = 4.31$

*L-Lysine yield (g/g $_{glucose}$) constant in all calculations
**L-Leucine (g/g $_{glucose}$)

Table A2. Reported microbial titer for some E8/B9 components

Compound	Titer (g/L)	Correction factor	Source

D-Calcium pantothenate	86.0	0.0751	[18,19]
FGF-2	2.00	3.23	[20]
Hypoxanthine	1.23	5.24	[21]
Insulin	4.00	1.62	[22]
Riboflavin	16.4	0.394	[23]
Transferrin	2.33	2.77	[24]
Vitamin B12	0.18	35.7	[25]

Calculation A3. Correction factor calculation example for compounds with reported titer

Insulin correction factor = 4.00 $g_{insulin}$ / 10 $g_{glucose}$ = 0.4 $g_{insulin}$ / $g_{glucose}$ → $0.646^{*}/0.4 = 1.62$

*L-Lysine yield (g/g $_{glucose}$) constant in all calculations

L-Arginine-HCl
Microbial L-arginine production is utilized as a substitute for L-arginine-HCl production. Additional resources with processing L-arginine into L-arginine-HCl-H_2O are not accounted for.

L-Asparagine-H_2O
L-Asparagine can be synthesized utilizing L-aspartic acid which is esterified followed by treatment with ammonia [4]. To estimate the embedded resources in asparagine production, the yield (.836 g/g glucose) for aspartic acid production was utilized. The resources utilized for esterification, ammonia treatment and additional steps for the conversion of L-asparagine to L-asparagine H_20 are not accounted for.

L-Cysteine-Cl-H_2O
Microbial L-cysteine production is utilized as a substitute for L-cysteine-HCl-H_2O production. Additional resources with processing L-cysteine into L-cysteine-HCl-H_2O are not accounted for.

L-Cystine
It is the oxidized dimer formed from a pair of cysteine molecules. The reported yield for the microbial production of cysteine is 0.06 g/g glucose and this value is utilized for cystine production [6].

L-Histidine-HCl-H_2O
Microbial L-histidine production is utilized as a substitute for L-histidine-HCl-H_2O production. Additional resources with processing L-histidine into L-histidine-HCl-H_2O are not accounted for.

Sodium Pyruvate
Microbial pyruvate production is utilized as a substitute for sodium pyruvate production. Additional resources with processing pyruvate into sodium pyruvate are not accounted for.

Pyridoxal-HCl and Pyridoxine-HCl
Pyridoxal HCl and pyridoxine HCl are forms of B_6 and have been produced microbially via recombinant *Sinorhizobium meliloti* [14,19]. A titer of 1.3 g/L of B_6 has been report and this titer was used to estimate yield based upon a minimal media containing 4 g of glucose/L [14,26]. The

estimated yield was utilized to estimate the embedded resources for both pyridoxal HCl and pyridoxine HCl production and additional processing was not accounted for.

Appendix C- Enzymatic

L-Aspartic acid

L-aspartic acid has been described as being produced enzymatically with yields of up to 0.95 (g/g fumaric acid) being reached while utilizing fumaric acid as a feedstock [27,28]. Fumaric acid can be produced utilizing glucose as feedstock with a yield of 0.88 g/g glucose [29]. To estimate embedded resources, the required mass of glucose was determined (~1.20 g glucose per g of L-aspartic acid produced). The embedded resources associated with the mass of glucose was then attributed to L-aspartic acid production.

Appendix D- Chemical

The following section provides the methodology utilized to estimate the environmental impact of E8/B9 components. Table A3 provides the E8/B9 precursor components for each chemical classified E8/B9 component and additional details related to the life cycle inventory methodology can be found in this subsequent section.

Table A3. Chemically manufactured E8/B9 components' life cycle inventory accounting methods and precursors components

E8/B9 component	Life cycle inventory method/s*	Precursor components used to produce E8/B9 component
Ascorbic acid 2-phosphate	sub	*Glucose*
Biotin	stoi	*cysteine, glucose,* others-not accounted for
Sodium selenite	stoi	Selenium dioxide, *sodium hydroxide*, selenium, *hydrochloric acid, soda ash,* sulfur dioxide, *sulfur*
Cupric sulfate	eco	Copper oxide, *sulfuric acid,* copper cathode
Ferric nitrate	stoi	Ferric nitrate, iron, nitric acid, iron ore concentrate
Ferrous sulfate	eco	N/A (only ferrous sulfate accounted for due to being a by-product)
Folic acid	N/A	Only water use estimated, others-not accounted for
Zinc sulfate	eco	*Sulfuric acid*, zinc oxide
Phenol Red	stoi, sub	O-sulfobenzoic acid, phenol, benzoic acid, *sulfuric acid*, cumene, *oxygen*, toluene, benzene, *propylene*
Putrescene 2HCl	stoi	Putrescine, *HCl, acrylonitrile*, hydrogen cyanide
Glycine	eco	*Ammonia*, chloroacetic acid, *sodium hydroxide*, acetic acid, *chlorine*, carbon monoxide, *methanol*
Choline chloride	stoi	Ethylene oxide*, HCl,* trimethylamine, ethylene, *oxygen, ammonia, methanol*
Niacinamide	stoi	3-cyanopyridine, 3-methylpyridine, *ammonia, oxygen*, acrolein, *propylene*
Thiamine hydrochloride	stoi, sub	Thiamine chloride, *HCl, Acrylonitrile*

sub- Substitution of acceptable component/s and ecoinvent database was utilized to estimate embedded resources.
stoi- Stoichiometry was utilized to determine required mass of components and ecoinvent database was utilized when datasets were available.
eco- Only ecoinvent datasets were utilized to quantify embedded resources
Compounds which are italicized are utilized in the production of multiple E8/B9 components and their embedded resources were account as well (see Table A5).
*Additional explanation of life cycle inventory accounting methods detailed below

AA2P (ascorbic acid 2-phosphate)

An ecoinvent dataset for ascorbic acid 2-phosphate was not reported and ascorbic acid was deemed as an acceptable substitute. The required mass of glucose, the major component utilized in ascorbic acid production was determined via E8/B9 concentration of ascorbic acid 2-

phosphate and ecoinvent database. The energy and material flows were determined from the ascorbic acid ecoinvent dataset and from the material/energy associated with glucose produced from corn (see raw ingredients: glucose section).

Biotin

Biotin can be synthesized from multiple precursors utilizing multi-step reactions (Bonrath et al., 2009; Casutt et al., 2011; de Clercq, 1997; Tang et al., 2020). Multiple reaction schemes have been reported to be used for the conversion of cysteine to biotin and we assume cysteine as the starting reactant [30]. We assumed a yield of 50% due to the multiple reaction steps with potential for loss and differences in molecular weight. The yield could be potentially lower due to inefficiencies in production. Adequate data were not available to estimate inputs/outputs during the multiple step conversion process. Cysteine can be produced microbially utilizing glucose as a feedstock and the embedded resources were determined utilizing the method described in the microbial methods section and appendix B [29]. The embedded resources associated with mass of glucose was then attributed to biotin. The reported embedded resource estimates for biotin production should be considered a minimum due to unavailability of input/output data related to energy and material flows during the production.

Sodium selenite

The following production route was chosen for sodium selenite, however sodium selenite can be prepared utilizing other methods [34]. This production route was chosen to minimize the additional compounds required for sodium selenite production. After stoichiometric calculations were complete, ecoinvent datasets associated with selenium production (selenium, soda ash, sulfur dioxide and hydrogen chloride) were utilized to quantify the some of the embedded resources in sodium selenite production. Sodium hydroxide, soda ash, sulfur dioxide and hydrogen chloride are utilized in production of this component and multiple E8/B9 components (See appendix G for complete list). Ecoinvent datasets were not available for sodium selenite and selenium dioxide production.

Equation A2. Selenium dioxide production

$Se + air + heat \rightarrow SeO_2 + \text{products of combustion}$

Assumption: No Se is lost during conversion of Se to SeO_2. Theoretical yield is utilized. Energy usage is not calculated for stoichiometric equations.

Equation A3. Sodium selenite production

$SeO_2 + 2\ NaOH \rightarrow Na_2SeO_3 + H_2O$

Assumption: Theoretical yield is utilized. Energy usage is not calculated for stoichiometric equations.

Cuperic Sulfate

Ecoinvent datasets were utilized to estimate material and energy flows associated with cuperic sulfate production (copper sulfate, copper oxide and copper cathode). Sulfuric acid is utilized in production of this component and multiple E8/B9 components (See appendix G) for related ecoinvent datasets. Copper cathode production data set includes embedded resources from copper mining.

Ferric Nitrate
Ferric nitrate can be prepared by adding nitric acid to iron pellets, powder or scrap iron [35]. After stoichiometric calculations were complete, ecoinvent datasets (Iron mining and beneficiation, iron pellet and nitric acid) were utilized to quantify the some of the embedded resources and outputs in ferric nitrate production. Ecoinvent data set was not available for ferric nitrate production.

Equation A4. Ferric nitrate production
$Fe + 4\ HNO_3 \rightarrow Fe(NO_3)_3 + NO + 2\ H_2O$
Assumption: Theoretical yield is utilized.

Ferrous sulfate
Ferrous sulfate is generally produced as a by-product of steel manufacture [36]. The utilized ecoinvent dataset (iron sulfate) accounts for this and only the embedded resources from additional refining is accounted for in the dataset and our model.

Folic acid
Chemical synthesis of folic acid largely occurs in an inexpensive, one-pot process (Mair et al., 2019). The economic viability of the chemical synthesis of folic acid largely prevents other production methods such as microbial fermentation or refinement from raw materials. Adequate data was not available to estimate embedded energy, but wastewater production was estimated in be 250-300 kg of wastewater for each kg of folic acid produced[38].

Zinc Sulfate
Ecoinvent data sets were utilized to estimate embedded resources and outputs in zinc sulfate production (zinc sulfate and zinc oxide). Sulfuric acid is utilized in production of this component and multiple E8/B9 components (See appendix G). The embedded resources for zinc scrap are not included due to being a by-product of other production process and iron scrap being utilized as a stand in the dataset.

Phenol red
Phenol red (phenolsulfophthalein) can be obtain by the condensation of o-sulfobenzoic acid anhydride with phenol [39]. Sulfobenzoic acid (mostly m-sulfobenzoic acid) derived from benzoic acid and sulfuric acid is used as a substitute for pure o-sulfobenzoic acid [40]. Stochiometric calculations were conducted to determine the mass of compound needed when information was unavailable in ecoinvent datasets. Ecoinvent datasets were utilized to estimate embedded resources and outputs in phenol red production (phenol, benzoic acid, toluene, and benzene). Sulfuric acid, propylene, oxygen, in ground natural gas and crude oil are utilized in production of this component and multiple E8/B9 components (See appendix G). Ecoinvent datasets were unavailable for phenol red and sulfobenzoic acid.

Putrescine-2HCl
Putrescine and HCl required mass and embedded resources were determined utilizing stochiometric calculations and ecoinvent datasets. Putrescine can be produced utilizing acrylonitrile and hydrogen cyanide in the presence of tertiary amine and with subsequent hydrogenation [41]. Ecoinvent datasets were utilized to estimate the material flows in Putrescine-

2HCl (Acrylonitrile, HCl, hydrogen cyanide). Both acrylonitrile and HCl are utilized in production of this component and multiple E8/B9 components (See appendix G). Ecoinvent datasets were unavailable for putrescine and putrescine-2HCl.

Glycine

Ecoinvent data sets were utilized to estimate energy and material flows during glycine production (glycine, chloroacetic acid, acetic acid, and carbon monoxide). Ammonia, chlorine (liquid), sodium hydroxide and methanol are utilized in the production of this component as well as multiple E8/B9 components (See section appendix G).

Choline Chloride

Choline chloride is produced via reaction between hydrochloric acid, trimethylamine and ethylene oxide (equation A5) [42]. Ecoinvent datasets were utilized to estimate embedded resources (ethylene oxide and trimethylamine). Hydrochloric acid, ethylene oxygen, ammonia, methanol, in ground natural gas, and crude oil are utilized in the production of this component as well as multiple E8/B9 components (See appendix G). An ecoinvent dataset was unavailable for choline chloride.

Equation A5. Choline chloride production

$$HCl + N(CH_3)_3 + C_2H_4O \rightarrow C_5H_{14}ClNO$$

Assumption: Theoretical yield is utilized.

Niacinamide

Niacinamide can be produced utilizing microbial synthesis with precursor compounds being produced chemically [43,44]. Niacinamide can be produced via microbial conversion of 3-cyanopyridine to niacinamide with a 94.5% yield [43]. 3-cyanopyridine can be produced via the ammoxidation of 3-methylpryidine (equation A6) [44]. Ecoinvent datasets were utilized to estimate embedded resources for niacinamide (3-methylprydine and acrolein). Ammonia, oxygen and propylene are utilized for the production of this component as well as multiple E8/B9 components (See appendix G). Embedded resources for niacinamide and 3-cyanopyridine production are not accounted.

Equation A6. 3-cyanopydrine production

$$H_3CC_5H_4N + NH_3 + 1.5\ O_2 \rightarrow NCC_5H_4N + 3\ H_2O$$

Assumptions: Theoretical yield is utilized. Stoichiometric calculation does not account for high density cell growth in bioreactor.

Thiamine hydrochloride

Thiamine hydrochloride can be manufactured by combining thiamine chloride with one molar equivalent of hydrochloric acid [45]. Thiamine production process was then utilized as substitute for thiamine chloride production process and acrylonitrile was utilized as the starting material for thiamine production [46]. Acrylonitrile and hydrochloric acid production was then utilized to estimate embedded resources in thiamine hydrochloride production. The embedded resources utilized during thiamine and the intermediates production processes are not included in the assessment.

Appendix E- Solvay

Sodium bicarbonate

Sodium bicarbonate production can be integrated into a soda ash production utilizing the Solvay process [47]. To produce one ton of sodium bicarbonate approximately 0.7 tonnes of raw soda ash and 550 kg of CO_2 (approximately 53% of the CO2 is released to atmosphere) are ulitilzed [47]. Equation A7 illustrates the theoretical mass balance to produce sodium bicarbonate. The CO_2 utilized for soda ash is considered to be a product of combustion and embedded resources for the CO_2 is not accounted.

Equation A7. Theoretical sodium bicarbonate production

$Na_2CO_3 + CO_2 + H_2O \rightarrow 2NaHCO_3$

Calcium chloride

Calcium chloride is assumed to be produced from the Solvay process and soda ash. The calcium chloride is assumed to dried and the embedded resources for liquor production and drying processes are accounted for [48]. The drying process can produce a product that is 75-82% flake or 100% prills [48]. The 100% prills production was assumed to be the product utilized for E8/B9 production. Table A3 provides estimates for embedded energy and water in use for calcium chloride production.

Table A3. Energy and water inputs for calcium chloride prills (100% w/w) production from soda process

Thermal energy for liquor production (GJ/ton)	7-9
Electrical energy for liquor production (GJ/ton)	0.3-0.5
Cooling water for liquor production (m^3/ton)	40
Thermal energy for prills production (GJ/ton)	7-9
Electrical energy for prills production (GJ/ton)	0.6-0.8
Cooling water for prills production (m^3/ton)	n/a

*Table developed from European Union reference document [48]

Sodium Phosphates

Sodium phosphates (monobasic and dibasic) are estimated to require approximately the same embedded resources and are not delineated between in embedded resource summation. Sodium phosphate production requires the use of soda ash or sodium hydroxide for production and are categorized as a product of the Solvay process due to soda ash being a major reactant. Ecoinvent datasets (sodium phosphate, purified phosphoric acid, phosphoric acid (fertilizer), quicklime, and beneficiated phosphate rock) were utilized to quantify the embedded resources in the sodium phosphate production process. Soda ash, sulfuric acid and crushed limestone are utilized for this component as well as multiple E8/B9 components (See section xx for related ecoinvent datasets).

Appendix F- Potash

Potassium Chloride
Potassium chloride is assumed to be produced from a mining operation and the ecoinvent dataset starts at the extraction at mine and ends with production of 1 kg of potassium chloride.

Magnesium Chloride
Magnesium chloride can be produced from a variety of sources including salt lakes, underground brines, residual brines from the potash industry [48]. The embedded resources were estimated utilizing industry supplied data as an estimation for embedded energy and water (Table A4) [49]. Energy and water usage calculated using reported water and energy intensities of products [49].

Table A4. Embedded water and energy in magnesium chloride production

Energy (MJ/kg)	4.9×10^{-1}
Water (m^3/kg)	4.7×10^{-3}

Magnesium Sulfate
Magnesium sulfate is assumed to be produced from a mining operation and the reported embedded resources and outputs in the ecoinvent dataset starts at the extraction at mine and ends with production of 1 kg of magnesium sulfate.

Appendix G- List of components used in production of multiple E8/B9 components

Table A5. Materials used to produce more than one E8/B9 ingredient

**Material used multiple times for E8/B9 ingredient production**	**E8/B9 ingredients**
Sodium bicarbonate	E8/B9 media ingredient*
Sodium hydroxide	Sodium selenite, Glycine
Soda ash	Sodium selenite, Glycine, Sodium bicarbonate, Sodium phosphates
Limestone	Sodium selenite, Glycine, Sodium bicarbonate, Sodium phosphates
Brine (NaCl)	Sodium selenite, Glycine, Sodium bicarbonate, Sodium phosphates
HCl	Putrescine-2HCl, Choline chloride, Thiamine hydrochloride
Chlorine (liquid)	Putrescine-2HCl, Choline chloride, Thiamine hydrochloride, Glycine
Chlorine (gas)	Putrescine-2HCl, Choline chloride, Thiamine hydrochloride, Glycine
NaCl (brine excluded)	E8/B9 media component, Putrescine-2HCl, Choline chloride, Thiamine hydrochloride, Glycine
Sulfuric acid	Copper sulfate, Zinc sulfate, Phenol red, Sodium phosphates
Sulfur	Sodium selenite Copper sulfate, Zinc sulfate, Phenol red, Sodium phosphates
Acrylonitrile	Putrescine-2HCl, Thiamine hydrochloride
Ammonia	Glycine, Choline chloride, Niacinamide, Putrescine-2HCl, Thiamine hydrochloride
Propylene	Phenol red, Niacinamide, Putrescine-2HCl, Thiamine hydrochloride
Methanol	Glycine, Choline chloride
Oxygen (liquid)	Phenol red, Choline chloride, Niacinamide,
High pressure natural gas	Ferric nitrate, Glycine, Choline chloride, Niacinamide, Putrescine-2HCl, Thiamine hydrochloride
In ground natural gas	Phenol red, Choline chloride, Niacinamide, Putrescine-2HCl, Thiamine hydrochloride
Crude oil	Phenol red, Choline chloride, Niacinamide, Putrescine-2HCl, Thiamine hydrochloride

Table excludes E8/B9 components whose embedded resources were quantified using raw/microbial/enzymatic methods described in the material and methods section.
*Used in DMEM/F12 basal media and as an addition to E8 basal media (one of the 7 components besides DMEM/F12 basal media)

Appendix H- Transportation
Ecoinvent datasets were utilized to account for the resources used for transportation across each components supply chain. These data sets were then entered into OpenLCA and the LCIA methods software package was utilized to determine the environmental impacts of transportation across the supply chain. Additional information related to the transportation of E8/B9 can be found in the following appendix subsections.

Raw ingredients

Glucose
Each market was utilized for each stage of production (dry maize to glucose). The total embedded energy related to corn/glucose transport was determined then the embedded energy used per kg of glucose was determined.

Linoleic acid
Refined vegetable oil market as a substitute in the ecoinvent dataset. The corn transport was not accounted for on based on the assumption that wet milling will be utilized and is onsite.

Microbial- Yield and titer

Glycine which is an amino acid produce in bulk and the market is reported in ecoinvent will be utilized as a substitute for the transportation. The total required glucose will also be accounted for utilizing transportation per kilogram of glucose.

Enzymatic, Aspartic acid

Glucose and it's embedded resources utilized for transportation was utilized to estimate the embedded energy related to aspartic acid transportation.

Chemical, Biotin

The embedded transportation energy from cysteine and glucose transport was utilized to estimate embedded transportation energy.

Appendix I- Sensitivity analysis

DMEM/F12 basal medium was found to be the most environmentally impactful component of both E8 and B9af in all impact categories. To further understand the drivers of these impacts, an additional sensitivity analysis was conducted to determine which of the DMEM/F12 basal medium components (>50) most influenced its environmental impact. The sensitivity analysis indicates that the glucose input is the most environmentally impactful DMEM/F12 component. This is due to the glucose concentration being orders of magnitude greater than most other inputs except sodium chloride and HEPES. A 25% increase in glucose concentration changed each TRACI 2.1 output by 6-20% and each cumulative energy demand output by 6-12% (See appendix figures A1.0 and A2.0). A 25% increase sodium bicarbonate or sodium chloride concentration increased some TRACI outputs by ~3% and cumulative energy demand outputs by

~2%. Looking at broader categories of inputs, a 25% increase in all amino acids increases CED and TRACI 2.1 outputs by ~15%. It should also be noted that HEPES concentration is greater than glucose concentration, but the HEPES environmental impacts are not accounted for due to a lack of manufacturing data.

Figure A1. Percentage of change in each TRACI impact category from a 25% increase in DMEM/F12 glucose concentration

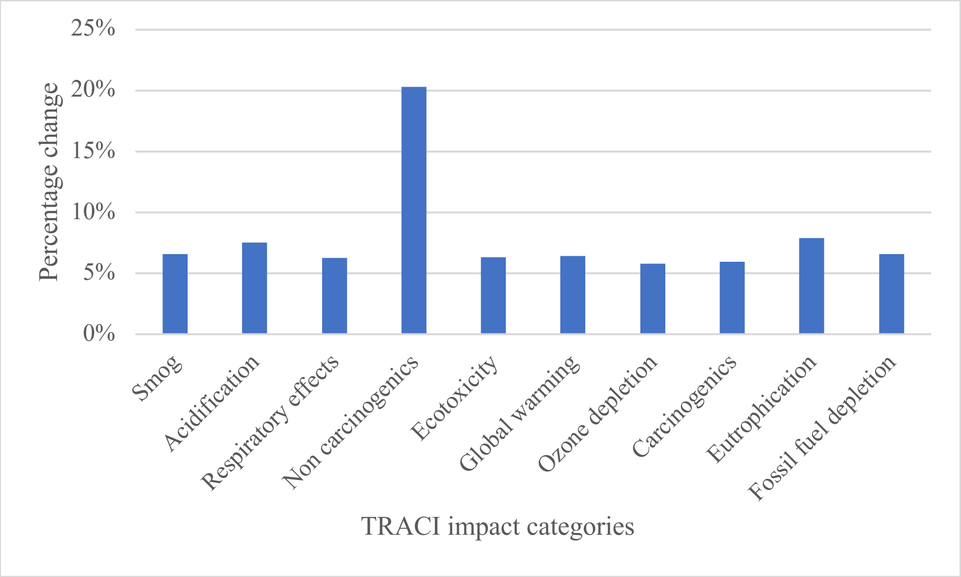

Figure A2. Percentage of change in each cumulative energy demand category from a 25% increase in DMEM/F12 glucose concentration

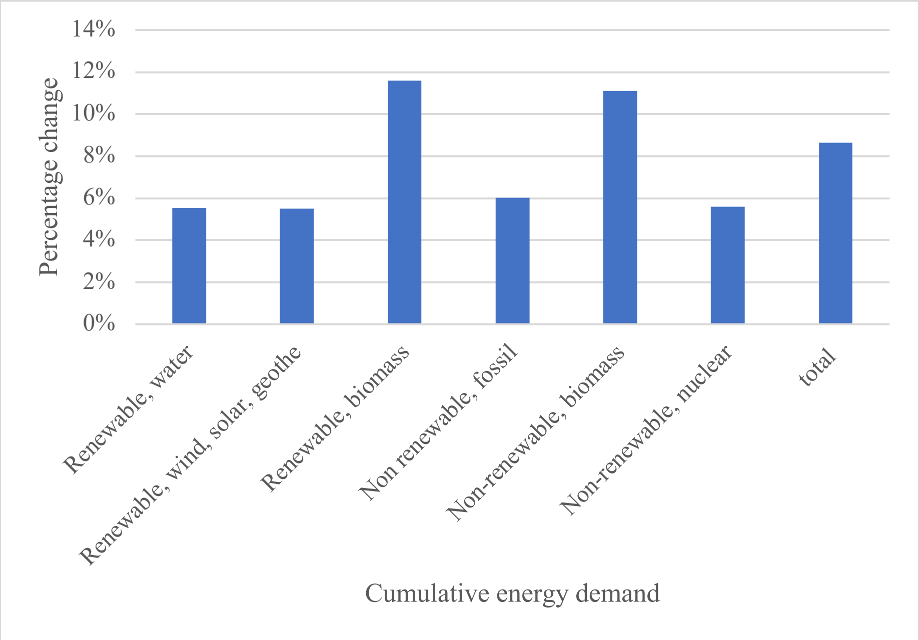

Supporting Material and appendices references

(1) ecoinvent Association. Ecoinvent Version 3.8 Database. ecoinvent association: Zurich, Switzerland 2021, p online. https://v38.ecoquery.ecoinvent.org/Home/Index.

(2) Matthäus, B. Oxidation of Edible Oils. *Oxidation in Foods and Beverages and Antioxidant Applications* **2010**, 183–238. https://doi.org/10.1533/9780857090331.2.183.

(3) Li, Y.; Han, P.; Wang, J.; Shi, T.; You, C. Production of Myo-Inositol: Recent Advance and Prospective. *Biotechnol Appl Biochem* **2021**. https://doi.org/10.1002/BAB.2181.

(4) Drauz, K.; Grayson, I.; Kleemann, A.; Krimmer, H.-P.; Leuchtenberger, W.; Weckbecker, C. Amino Acids. In *Ullmann's Encyclopedia of Industrial Chemistry*; American Cancer Society: Weinheim, Germany, 2007. https://doi.org/10.1002/14356007.A02_057.PUB2.

(5) Man, Z.; Xu, M.; Rao, Z.; Guo, J.; Yang, T.; Zhang, X.; Xu, Z. Systems Pathway Engineering of Corynebacterium Crenatum for Improved L-Arginine Production. *Scientific Reports 2016 6:1* **2016**, *6* (1), 1–10. https://doi.org/10.1038/srep28629.

(6) Liu, H.; Fang, G.; Wu, H.; Li, Z.; Ye, Q. L-Cysteine Production in *Escherichia Coli* Based on Rational Metabolic Engineering and Modular Strategy. *Biotechnol J* **2018**, *13* (5), 1700695. https://doi.org/10.1002/biot.201700695.

(7) Wen, J.; Bao, J. Engineering Corynebacterium Glutamicum Triggers Glutamic Acid Accumulation in Biotin-Rich Corn Stover Hydrolysate. *Biotechnology for Biofuels 2019 12:1* **2019**, *12* (1), 1–11. https://doi.org/10.1186/S13068-019-1428-5.

(8) Schwentner, A.; Feith, A.; Münch, E.; Stiefelmaier, J.; Lauer, I.; Favilli, L.; Massner, C.; Öhrlein, J.; Grund, B.; Hüser, A.; Takors, R.; Blombach, B. Modular Systems Metabolic Engineering Enables Balancing of Relevant Pathways for L-Histidine Production with Corynebacterium Glutamicum 06 Biological Sciences 0601 Biochemistry and Cell Biology. *Biotechnol Biofuels* **2019**, *12* (1), 65. https://doi.org/10.1186/s13068-019-1410-2.

(9) Félix, F. K. do C.; Letti, L. A. J.; Vinícius de Melo Pereira, G.; Bonfim, P. G. B.; Soccol, V. T.; Soccol, C. R. L-Lysine Production Improvement: A Review of the State of the Art and Patent Landscape Focusing on Strain Development and Fermentation Technologies. *Critical Reviews in Biotechnology*. Taylor and Francis Ltd November 17, 2019, pp 1031–1055. https://doi.org/10.1080/07388551.2019.1663149.

(10) Zhou, H.-Y.; Wu, W.-J.; Niu, K.; Xu, Y.-Y.; Liu, Z.-Q.; Zheng, Y.-G. Enhanced L-Methionine Production by Genetically Engineered Escherichia Coli through Fermentation Optimization. *3 Biotech* **2019**, *9* (3), 96. https://doi.org/10.1007/S13205-019-1609-8.

(11) Wendisch, V. F.; Jorge, J. M. P.; Pérez-García, F.; Sgobba, E. Updates on Industrial Production of Amino Acids Using Corynebacterium Glutamicum. *World J Microbiol Biotechnol* **2016**, *32* (6), 1–10. https://doi.org/10.1007/s11274-016-2060-1.

(12) Zhang, X.; Xu, G.; Shi, J.; Koffas, M. A. G.; Xu, Z. Microbial Production of L-Serine from Renewable Feedstocks. *Trends Biotechnol* **2018**, *36* (7), 700–712. https://doi.org/10.1016/j.tibtech.2018.02.001.

(13) Lütke-Eversloh, T.; Santos, C. N. S.; Stephanopoulos, G. Perspectives of Biotechnological Production of L-Tyrosine and Its Applications. *Applied Microbiology and Biotechnology*. Springer December 30, 2007, pp 751–762. https://doi.org/10.1007/s00253-007-1243-y.

(14) Wang, Y.; Liu, L.; Jin, Z.; Zhang, D. Microbial Cell Factories for Green Production of Vitamins. *Front Bioeng Biotechnol* **2021**, *0*, 473. https://doi.org/10.3389/FBIOE.2021.661562.

(15) Miyata, R.; Yonehara, T. Breeding of High-Pyruvate-Producing Torulopsis Glabrata with Acquired Reduced Pyruvate Decarboxylase. *J Biosci Bioeng* **1999**, *88* (2), 173–177. https://doi.org/10.1016/S1389-1723(99)80197-2.

(16) Li, Y.; Chen, J.; Lun, S. Biotechnological Production of Pyruvic Acid. *Appl Microbiol Biotechnol* **2001**, *57* (4), 451–459. https://doi.org/10.1007/S002530100804.

(17) Lee, H. C.; Kim, J. S.; Jang, W.; Kim, S. Y. High NADPH/NADP+ Ratio Improves Thymidine Production by a Metabolically Engineered Escherichia Coli Strain. *J Biotechnol* **2010**, *149* (1–2), 24–32. https://doi.org/10.1016/j.jbiotec.2010.06.011.

(18) Hohmann, H.-P.; Dijl, J. M. van; Krishnappa, L.; Prágai, Z. Host Organisms: Bacillus Subtilis. In *Industrial Biotechnology: Microorganisms*; Witmann, C., Liao, J., Eds.; John Wiley & Sons, Ltd, 2017; pp 221–297. https://doi.org/10.1002/9783527807796.CH7.

(19) Acevedo-Rocha, C. G.; Gronenberg, L. S.; Mack, M.; Commichau, F. M.; Genee, H. J. Microbial Cell Factories for the Sustainable Manufacturing of B Vitamins. *Curr Opin Biotechnol* **2019**, *56*, 18–29. https://doi.org/10.1016/J.COPBIO.2018.07.006.

(20) Sauer, D. G.; Mosor, M.; Frank, A. C.; Weiß, F.; Christler, A.; Walch, N.; Jungbauer, A.; Dürauer, A. A Two-Step Process for Capture and Purification of Human Basic Fibroblast Growth Factor from E. Coli Homogenate: Yield versus Endotoxin Clearance. *Protein Expr Purif* **2019**, *153*, 70–82. https://doi.org/10.1016/j.pep.2018.08.009.

(21) Liu, M.; Fu, Y.; Gao, W.; Xian, M.; Zhao, G. Highly Efficient Biosynthesis of Hypoxanthine in Escherichia Coli and Transcriptome-Based Analysis of the Purine Metabolism. *ACS Synth Biol* **2020**, *9* (3), 525–535. https://doi.org/10.1021/acssynbio.9b00396.

(22) Baeshen, N. A.; Baeshen, M. N.; Sheikh, A.; Bora, R. S.; Ahmed, M. M. M.; Ramadan, H. A. I.; Saini, K. S.; Redwan, E. M. Cell Factories for Insulin Production. *Microb Cell Fact* **2014**, *13* (1), 141. https://doi.org/10.1186/s12934-014-0141-0.

(23) Liu, S.; Hu, W.; Wang, Z.; Chen, T. Production of Riboflavin and Related Cofactors by Biotechnological Processes. *Microb Cell Fact* **2020**, *19* (1), 1–16. https://doi.org/10.1186/s12934-020-01302-7.

(24) Finnis, C. J. A.; Payne, T.; Hay, J.; Dodsworth, N.; Wilkinson, D.; Morton, P.; Saxton, M. J.; Tooth, D. J.; Evans, R. W.; Goldenberg, H.; Scheiber-Mojdehkar, B.; Ternes, N.; Sleep, D. High-Level Production of Animal-Free Recombinant Transferrin from Saccharomyces Cerevisiae. *Microb Cell Fact* **2010**, *9* (1), 87. https://doi.org/10.1186/1475-2859-9-87.

(25) Li, K.; Peng, W.; Zhou, J.; wei, S.; Cheng, X. Establishment of Beet Molasses as the Fermentation Substrate for Industrial Vitamin B12 Production by Pseudomonas Denitrificans. *Journal of Chemical Technology & Biotechnology* **2013**, *88* (9), 1730–1735. https://doi.org/10.1002/jctb.4025.

(26) King, J. *Minimal media*. http://web.mit.edu/king-lab/www/cookbook/rminimal.htm (accessed 2021-10-26).

(27) Yukawa, H.; Ookino, S.; Inui, M. L-Aspartic Acid, Production Processes. In *Encyclopedia of Industrial Biotechnology*; American Cancer Society, 2010; pp 1–3. https://doi.org/10.1002/9780470054581.EIB383.

(28) Appleton, H.; Rosentrater, K. A. Sweet Dreams (Are Made of This): A Review and Perspectives on Aspartic Acid Production. *Fermentation 2021, Vol. 7, Page 49* **2021**, *7* (2), 49. https://doi.org/10.3390/FERMENTATION7020049.

(29) Martin-Dominguez, V.; Estevez, J.; Ojembarrena, F. D. B.; Santos, V. E.; Ladero, M. Fumaric Acid Production: A Biorefinery Perspective. *Fermentation 2018, Vol. 4, Page 33* **2018**, *4* (2), 33. https://doi.org/10.3390/FERMENTATION4020033.

(30) de Clercq, P. J. Biotin: A Timeless Challenge for Total Synthesis. *Chem Rev* **1997**, *97* (6), 1755–1792. https://doi.org/10.1021/CR950073E.

(31) Bonrath, W.; Karge, R.; Netscher, T.; Roessler, F.; Spindler, F. Biotin - the Chiral Challenge. *Chimia (Aarau)* **2009**, *63* (5), 265–269. https://doi.org/10.2533/CHIMIA.2009.265.

(32) Tang, P.; Nie, B.; Huang, J.; Zhang, Y.; Zhang, J.; Chen, F.-E. Recent Advances of Pharmaceutical Process Chemistry and Its Innovation in China: Part 1. *Pharmaceut Fronts* **2020**, *2*, 28–54. https://doi.org/10.1055/s-0040-1701652.

(33) Casutt, M.; Koppe, T.; Schwarz, M. Vitamins, 9. Biotin. *Ullmann's Encyclopedia of Industrial Chemistry* **2011**. https://doi.org/10.1002/14356007.O27_O12.

(34) Brauer, G. Selenium. In *Handbook of Preparative Inorganic Chemistry*; Academic Press Inc.: London, 1963; Vol. 1, pp 415–436.

(35) National Center for Biotechnology. PubChem Compound Summary for CID 25251, Ferric Nitrate. *PubChem*. 2021. https://pubchem.ncbi.nlm.nih.gov/compound/Ferric-nitrate (accessed 2021-10-20).

(36) Wildermuth, E.; Stark, H.; Friedrich, G.; Ebenhöch, F. L.; Kühborth, B.; Silver, J.; Rituper, R. Iron Compounds. In *Ullmann's Encyclopedia of Industrial Chemistry*; American Cancer Society, 2000; p online. https://doi.org/10.1002/14356007.A14_591.

(37) Mair, P.; Péter, S.; Stemmler, R. T.; Wehrli, C.; Weimann, B.-J.; Hengartner, U.; Saizieu, A. Vitamins, 12. Vitamin B9. *Ullmann's Encyclopedia of Industrial Chemistry* **2019**, 1–15. https://doi.org/10.1002/14356007.O27_O13.PUB2.

(38) Bryne, J. *New capacity set to heighten Chinese folic acid price slide*. Feednavigator.com. https://www.feednavigator.com/Article/2015/10/20/New-capacity-set-to-heighten-Chinese-folic-acid-price-slide (accessed 2022-01-09).

(39) Gessner, T.; Mayer, U. Triarylmethane and Diarylmethane Dyes. In *Ullmann's Encyclopedia of Industrial Chemistry*; Wiley-VCH Verlag GmbH & Co. KGaA, 2011; p online. https://doi.org/10.1002/14356007.A27_179.

(40) Reese, J. Orienting Influences in the Benzene Ring. The Sulfonation of Benzoic Acid. *J Am Chem Soc* **1932**, *54*, 2009–2017.

(41) Broadwith, P. Putrescine. *Chemistry World*. Royal Society of Chemistry: London February 15, 2011, p online.

(42) Johnson Matthey Davy Technologies. *Choline chloride*. Licensed Processes. https://web.archive.org/web/20140918142932/http://www.davyprotech.com/what-we-do/licensed-processes-and-core-technologies/licensed-processes/choline-chloride/specification/ (accessed 2021-10-21).

(43) Wang, Z.; Liu, Z.; Cui, W.; Zhou, Z. Establishment of Bioprocess for Synthesis of Nicotinamide by Recombinant Escherichia Coli Expressing High-Molecular-Mass Nitrile Hydratase. *Applied Biochemistry and Biotechnology 2017 182:4* **2017**, *182* (4), 1458–1466. https://doi.org/10.1007/S12010-017-2410-Y.

(44) Shimizu, S.; Watanabe, N.; Kataoka, T.; Shoji, T.; Abe, N.; Morishita, S.; Ichimura, H. Pyridine and Pyridine Derivatives. In *Ullmann's Encyclopedia of Industrial Chemistry*; American Cancer Society, 2000. https://doi.org/10.1002/14356007.A22_399.

(45) ChEBI. Thiamine Hydrochloride. *Chemical Entities of Biological Interest*. Elixir core data resource 2021, p online. https://www.ebi.ac.uk/chebi/searchId.do?chebiId=CHEBI:49105 (accessed 2021-10-21).
(46) Létinois, U.; Moine, G.; Hohmann, H.-P. Vitamin B1 (Thiamin). In *Ullmann's Encyclopedia of Industrial Chemistry*; Wiley-VCH, 2020; p online. https://doi.org/10.1002/14356007.O27_O09.PUB2.
(47) European Commission. Soda Ash. In *Integrated Pollution Prevention and Control Reference Document on Best Available Techniques for the Manufacture of Large Volume Inorganic Chemical Solids and Other Industry*; 2007; pp 31–103.
(48) European Commission. Selected Illustrative LVIC-S Industry Products. In *Integrated Pollution Prevention and Control Reference Document on Best Available Techniques for the Manufacture of Large Volume Inorganic Chemical Solids and Other Industry*; 2007; pp 343–593.
(49) Compass minerals. *Sustainability Report 2016*; Overland park, 2016. https://www.compassminerals.com/sustainability-report-2016/environmental-responsibility.html#environmental-targets (accessed 2021-10-22).
